# Premature activation of Cdk1 leads to mitotic events in S phase and embryonic lethality

**DOI:** 10.1038/s41388-018-0464-0

**Published:** 2018-09-06

**Authors:** Radoslaw Szmyd, Joanna Niska-Blakie, M. Kasim Diril, Patrícia Renck Nunes, Konstantinos Tzelepis, Aurélie Lacroix, Noémi van Hul, Lih-Wen Deng, Joao Matos, Oliver Dreesen, Xavier Bisteau, Philipp Kaldis

**Affiliations:** 1 Institute of Molecular and Cell Biology (IMCB), A*STAR (Agency for Science, Technology and Research), 61 Biopolis Drive, Proteos #3-09, Singapore, 138673 Republic of Singapore; 2 National University of Singapore (NUS), NUS Graduate School for Integrative Sciences and Engineering, Singapore, 117597 Republic of Singapore; 3 Bioinformatics Institute (BII), A*STAR, Singapore, 138671 Republic of Singapore; 4 National University of Singapore (NUS), Department of Biochemistry, Singapore, 117597 Republic of Singapore; 5 ETH Zürich, Department of Biology, Zürich, Switzerland; 6 Institute of Medical Biology, A*STAR, Singapore, 138648 Republic of Singapore; 7 Present Address: Izmir Biomedicine and Genome Institute, Dokuz Eylul University, 35340 Izmir, Turkey; 8 Present Address: Ecole Polytechnique Federale de Lausanne, School of Life Sciences, CH-1015 Lausanne, Switzerland; 9 Present Address: Wellcome Trust Sanger Institute, Hinxton, Cambridge CB10 1SA UK; 10 Present Address: Department of Chemistry and Centre for Self-Assembled Chemical Structures (CSACS), McGill University, 801 Sherbrooke Street West, Montreal, QC H3A 0B8 Canada

**Keywords:** Cancer models, Mitosis

## Abstract

Cell cycle regulation, especially faithful DNA replication and mitosis, are crucial to maintain genome stability. Cyclin-dependent kinase (CDK)/cyclin complexes drive most processes in cellular proliferation. In response to DNA damage, cell cycle surveillance mechanisms enable normal cells to arrest and undergo repair processes. Perturbations in genomic stability can lead to tumor development and suggest that cell cycle regulators could be effective targets in anticancer therapy. However, many clinical trials ended in failure due to off-target effects of the inhibitors used. Here, we investigate in vivo the importance of WEE1- and MYT1-dependent inhibitory phosphorylation of mammalian CDK1. We generated *Cdk1*^*AF*^ knockin mice, in which two inhibitory phosphorylation sites are replaced by the non-phosphorylatable amino acids T14A/Y15F. We uncovered that monoallelic expression of CDK1^AF^ is early embryonic lethal in mice and induces S phase arrest accompanied by γH2AX and DNA damage checkpoint activation in mouse embryonic fibroblasts (MEFs). The chromosomal fragmentation in *Cdk1*^*AF*^ MEFs does not rely on CDK2 and is partly caused by premature activation of MUS81-SLX4 structure-specific endonuclease complexes, as well as untimely onset of chromosome condensation followed by nuclear lamina disassembly. We provide evidence that tumor development in liver expressing CDK1^AF^ is inhibited. Interestingly, the regulatory mechanisms that impede cell proliferation in CDK1^AF^ expressing cells differ partially from the actions of the WEE1 inhibitor, MK-1775, with p53 expression determining the sensitivity of cells to the drug response. Thus, our work highlights the importance of improved therapeutic strategies for patients with various cancer types and may explain why some patients respond better to WEE1 inhibitors.

## Introduction

Proliferation of eukaryotic cells requires faithful replication and segregation of the genetic material during cell cycle progression and mitosis. Timely progression through these events is regulated by cyclin-dependent kinases (CDKs). Interestingly, CDK1 alone is sufficient to drive mouse embryogenesis until midgestation in the absence of interphase CDKs (CDK2/3/4/6) [1], whereas CDK1 loss leads to early embryonic lethality due to mitosis entry failure [2]. CDK activity is temporally and spatially regulated through the cell cycle and requires binding to cyclin partners [3], as well as posttranslational modifications. Once associated with cyclin A2 or B1, CDK1 activity is further controlled through its inhibitory phosphorylation on threonine 14 (T14 [4–6]), tyrosine 15 (Y15 [4, 7]), and its activating phosphorylation on threonine 161 (T161[8, 9]). WEE1-dependent phosphorylation on the Y15 residue prevents unscheduled entry into mitosis by keeping CDK1/cyclin B1 complexes in a low activity state [10–13]. In metazoans, MYT1-dependent modification of T14 precludes premature activation of CDK1/cyclin B1 complexes right before their nuclear translocation [4, 6, 13, 14]. The dual-specificity phosphatase CDC25 regulates the mitotic onset by the abrupt dephosphorylation of T14 and Y15, triggering complete activation of CDK1/cyclin B1 complexes [15–17]. Therefore, the dephosphorylation of CDK1 on its inhibitory residues is believed to be a rate-limiting step for its activation and entry into mitosis [18, 19].

As expected, overexpression of CDK1, as well as high expression of its binding partner cyclin B1 [20, 21], has been described in many cancers with poor prognosis. Altered activity of CDK1 due to deranged p53 and the DNA damage-signaling pathway has been reported [22–29]. In response to DNA damage, induction of CHK1/CHK2 controls CDK1 activity via WEE1 and CDC25 regulation to ensure complete DNA repair before entry into mitosis [26, 30]. As the DNA damage response (DDR) is suppressed by elevated CDK1 activity, triggering premature mitotic events in combination with DNA-damaging agents has become an attractive therapeutic strategy for cancer patients [31–33]. Based on this, WEE1 inhibition is an effective approach in clinical trials [29, 33]. However, its off-target effects and cross-inhibition of other kinases (like CDK2) needs further research to establish an effective anticancer strategy [34–38]. The assessment of premature CDK1 activity using diverse approaches has resulted in variable results. WEE1 itself is essential for proliferation and embryogenesis in mice [39] and its inhibition/silencing induces DNA damage but also mitotic catastrophe [29, 34] through premature activation of several substrates including the structure selective endonuclease (SSE) MUS81 [34, 40, 41] in multiple cell lines. In contrast, the ectopic expression of mutant CDK1^T14A/Y15F^ (hereafter referred to as CDK1^AF^) alone in mammalian cell lines has rather insignificant effects on S phase progression and mitotic timing but when co-expressed with cyclin B1, greatly increases the frequency of premature mitotic events [11, 42, 43]. In addition, the levels of the overexpressed mutant CDK1^AF^ in presence or absence of wild-type CDK1 affects the biological outcome [44].

To fully understand the impact of aberrant CDK1 activity and the importance of the inhibitory phosphorylation on T14 and Y15 at the endogenous level in vivo, we have taken advantage of our recently generated *Cdk1*^*AF*^ knockin mouse model, in which both inhibitory phosphorylation sites are replaced by non-phosphorylatable amino acids, T14A and Y15F [45]. We observed that monoallelic expression of *Cdk1*^*AF*^ leads to early embryonic lethality and is associated with altered activation of key cell cycle regulators, premature mitotic events, increased levels of DNA damage, replication stress and chromosomal fragmentation leading to S phase failure. We provide evidence of the involvement of MUS81 in these defects, which indicates that inhibitory phosphorylation of CDK1 during S phase safeguards genomic integrity by protecting chromatin from unscheduled endonucleolytic digestion by the mitotic MUS81-SLX4 complexes. Moreover, our work unravels the importance of the p53 status for the sensitivity of cells to CDK1 inhibitory phosphorylation, both in *Cdk1*^*AF*^ and control cells treated with the WEE1 inhibitor, MK-1775. Last but not least, we show that liver expressing mutant CDK1^AF^ protein does not develop tumors unlike control mice after induction of tumorigenesis.

## Results

### The expression of CDK1^AF^ leads to lethality accompanied by DNA damage in mice

**Fig. 1:**
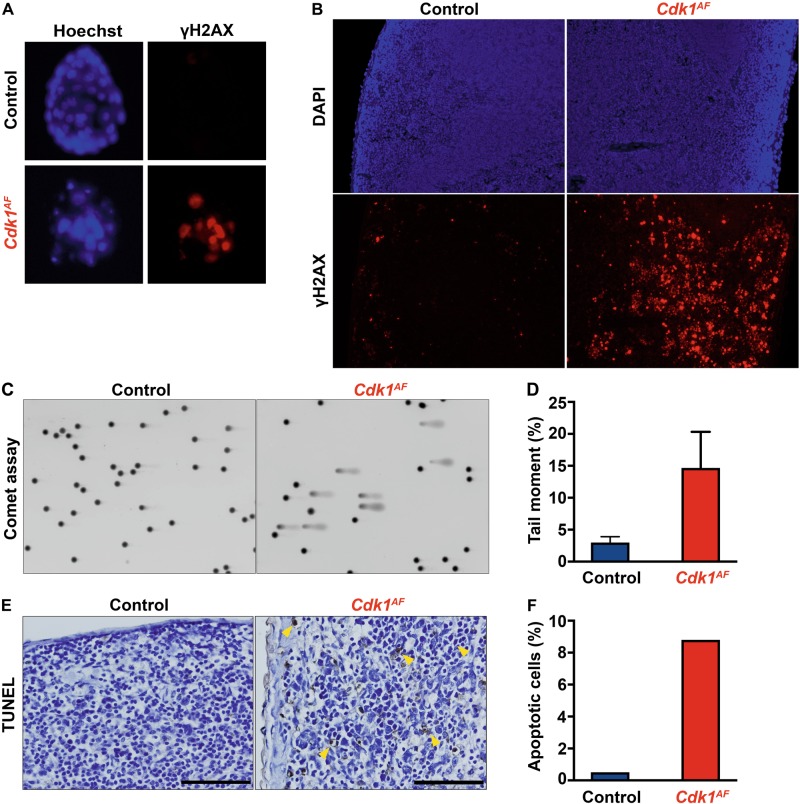
The expression of CDK1^AF^ leads to early embryonic lethality. **a** Control (*Cdk1*^*+/SAF*^) and β-actin-Cre *Cdk1*^*+/AF*^ blastocysts were visualized with Hoechst staining (nuclei). The level of DNA damage was assessed through immunofluorescence staining of phospho-H2AX. **b** The expression of *Cdk1*^*+/AF*^ was induced in all tissues of adult Rosa26-CreER^T2^ mice upon tamoxifen IP administration. Spleen sections were stained for phospho-γH2AX to visualize DNA damage response. **c** DNA damage in spleen of control and Rosa26-CreER^T2^ mutant mice was analyzed by Comet assays using the tail moment as a parameter to determine the extent of DNA breaks. **d** Quantification of DNA breaks was calculated based on the tail moment. **e** To investigate chromosomal fragmentation, spleen sections from tamoxifen injected control and *Cdk1*^*+/AF*^ Rosa26-CreER^T2^ mice were analyzed by TUNEL assay to evaluate apoptosis. Yellow arrows indicate apoptotic cells with extensive DNA breaks. **f** The number of apoptotic cells was quantified in relation to the total number of cells

To investigate the consequences of CDK1^AF^ expression in vivo, we crossed *Cdk1*^*+/SAF*^ (hereafter referred to as *Cdk1*^*SAF*^; SAF stands for Stop-AF [45], whereas AF refers to the AF allele that is actively expressed) animals with mice ubiquitously expressing β-actin-Cre and analyzed the progeny at different developmental stages. Resulting *Cdk1*^*AF*^ P21 pups and E13.5 embryos were not viable, whereas mutant blastocysts (E3.5) were obtained at expected frequency (Table 1). Compared with controls, *Cdk1*^*AF*^ blastocysts displayed a reduced number of cells accompanied with an increase in the phosphorylation on S139 of the H2AX histone variant (hereafter called γH2AX) (Fig. 1a). To further examine the effects of the ubiquitous CDK1^AF^ expression in adult mice, we injected tamoxifen in *Cdk1*^*SAF*^ animals harboring the Rosa26-CreER^T2^ transgene [46] (hereafter referred to as Rosa-Cre). Similarly to what we previously observed for *Cdk1* knockout adult mice [2], animals expressing CDK1^AF^ died within 5–6 days after tamoxifen administration, indicating that CDK1^AF^ expression is also lethal in adult animals. Spleen of control and mutant animals was collected 4 days after tamoxifen injection to evaluate the extent of the DNA damage. Staining for γH2AX of spleen (Fig. 1b) and other tissue sections (data not shown) from *Cdk1*^*AF*^ mice revealed a prominent signal increase compared with control mice. Comet assays on splenocytes from *Cdk1*^*AF*^ mice confirmed the observed increase of DNA damage since the tail moment was 14 times higher than in the control animals (Fig. 1c, d). In order to assess whether CDK1^AF^ expression could lead to apoptosis, we performed terminal deoxynucleotidyl transferase dUTP nick end labeling (TUNEL) assays on spleen sections from control and CDK1^AF^-expressing mice. Less than 1% apoptotic cells were observed in wild-type mice, whereas in *Cdk1*^*AF*^ mice >8% were detected (Fig. 1e; yellow arrows, Fig. 1f). These in vivo observations suggest that the timely control of CDK1 activity via its inhibitory phosphorylation on T14 and Y15 is essential during the embryogenesis and adult life to prevent the formation of DNA breaks and the onset of apoptosis.

**Table 1:** *Cdk1*^*+/AF*^ mice are early embryonic lethal

Age	Total	Control	*Cdk1* ^*AF*^
P21 pups	39	39	0
E13.5 embryos	47	47	0
E3.5 blastocysts	33	25 (76%)	8 (24%)

### S phase failure in *Cdk1*^*AF*^ MEFs

**Fig. 2:**
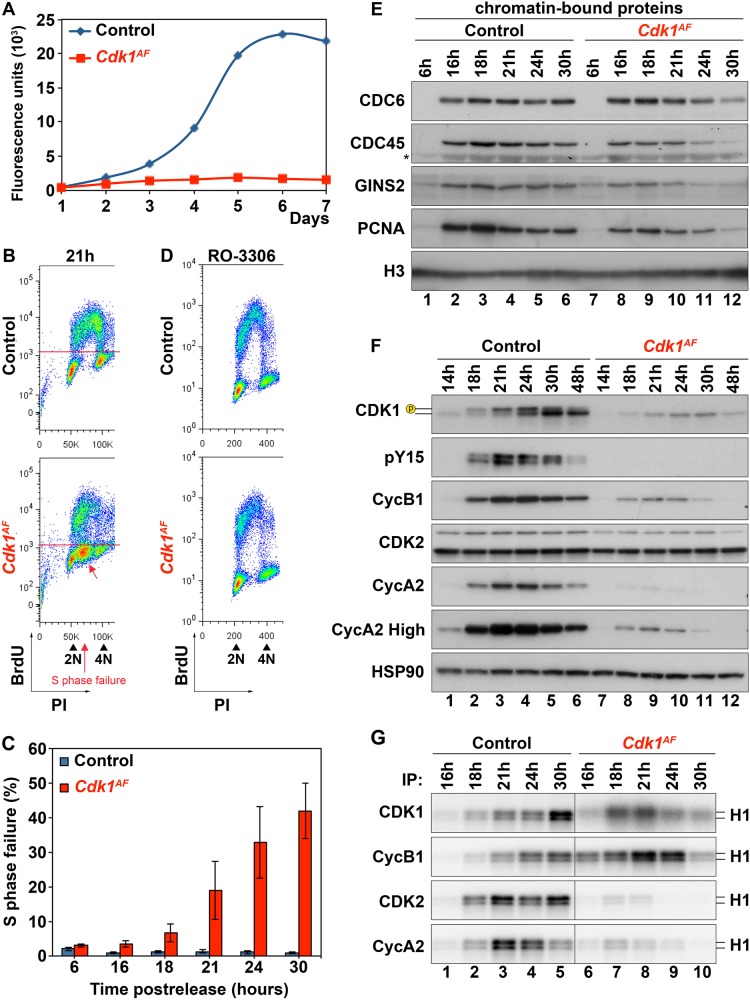
Characterization of *Cdk1*^*AF*^ MEFs. Primary MEFs were synchronized at G_0_/G_1_ by serum starvation as described in Materials and methods section. The expression of CDK1^AF^ was induced during the starvation period. **a** The proliferative potential of *Cdk1*^*flox/SAF*^ (control) and *Cdk1*^*null/AF*^ (*Cdk1*^*AF*^) MEFs was monitored by alamarBlue proliferation assay for seven days. **b** The distribution of cells at 21-h time point after release from serum starvation was analyzed by BrdU FACS. DNA content was determined by propidium iodide (PI) staining. Based on BrdU incorporation and DNA content cells were classified into four categories: G_1_, S, G_2_/M and S phase failure. **c** Quantitative analysis of S phase failure from (**b**). Data are represented as mean ± SD from three independent experiments. **d** BrdU FACS analysis of *Cdk1*^*+/SAF*^ (control) and *Cdk1*^*+/AF*^ MEFs (*Cdk1*^*AF*^) at 24 h after release in the absence or presence of 10 µM of CDK1 inhibitor, RO-3306. **e** The abundance of chromatin-bound proteins was determined in *Cdk1*^*flox/SAF*^ and *Cdk1*^*null/AF*^ MEFs by cellular fractionation followed by western blotting with the indicated antibodies. Histone H3 served as a loading control. **f** Protein extracts from *Cdk1*^*flox/SAF*^ and *Cdk1*^*null/AF*^ MEFs were subjected to western blotting using the indicated antibodies. HSP90 served as a loading control. **g** Protein extracts from (**d**) were subjected to immunoprecipitation with the indicated antibodies. Kinase activities of immunoprecipitates were measured by in vitro kinase assays, in which radioactive ATP and histone H1 were used as substrates. Results are representative of two (**e**)/three (**f**–**g**) independent experiments. * unspecific band; p – phosphorylated form of Cdk1

To unravel the molecular mechanism of *Cdk1*^*AF*^-induced lethality, we used mouse embryonic fibroblasts (MEFs) expressing 4-hydroxytamoxifen (4-OHT) inducible Esr1-CreER^T2^ to induce CDK1^AF^ expression. As displayed in Fig. 2a, the proliferation of *Cdk1*^*AF*^ MEFs over 7 days was impaired once released after synchronization at G_0_/G_1_ by serum starvation. Fluorescence-activated cell sorting (FACS) analysis of BrdU-labeled cells after release from serum starvation revealed the appearance of an intermediate BrdU-negative population located between the G_1_ and G_2_ population of mutant cells (Fig. 2b and S1A, red arrow). Both control and *Cdk1*^*AF*^ MEFs were able to enter S phase and initiated replication at 16 h after release (Figure S1A). Over a period of 30 h, during which control MEFs successfully duplicated their genome and divided (Figure S1A), *Cdk1*^*AF*^ cells displayed an increasing population with partially replicated DNA and never completed DNA replication. The percentage of *Cdk1*^*AF*^ cells forming the intermediate population, which could be interpreted as unfinished S phase or S phase failure, gradually increased from 4% at 16 h to 42 % at the 30-h time point (Fig. 2c). The lack of proliferation indicated that the S phase cell cycle arrest in *Cdk1*^*AF*^ MEFs was permanent.

Our past work suggests that the loss of either CDK2 or CDK1 activity does not have major effects on S phase progression [2, 47]. To investigate whether a reduction of CDK1 activity could rescue *Cdk1*^*AF*^-induced defects, we treated MEFs with the specific CDK1 inhibitor, RO-3306 [48]. The low dose of RO-3306 used had no effect on S phase progression in control cells but rescued S phase failure in *Cdk1*^*AF*^ MEFs (Fig. 2d, S1B). This indicates that the unscheduled CDK1 activity is the cause of the impaired cell cycle progression in mutant fibroblasts.

However, the molecular pathway leading to S phase failure in mutant *Cdk1*^*AF*^ cells still remains unknown. To determine its origin, we first investigated the initiation of DNA replication by assessing the formation and loading of the prereplication complex (pre-RC) and its transformation into the preinitiation complex (pre-IC). The pre-RC consists of a double hexameric MCM complex, ORC, CDC6 and CDT1 proteins [49–55]. The pre-RC is loaded at replication origins during G_1_ phase. Once cells enter S phase, the pre-RC is converted to active helicase/replisome pre-IC in a DDK/CDK-dependent manner by recruiting CDC45 [56] followed by GINS2 loading induced by CDK2/cyclin A activity [57, 58]. We assessed the abundance of different replisome components recruited to chromatin using cellular fractionation followed by western blotting (Fig. 2e). None of the replication factors were detected on chromatin in control and mutant cells in the early G_1_ phase (6 h). This is likely due to the low expression levels after serum starvation, as these proteins were also undetected in the soluble fraction (Figure S1C). By 16 h, pre-RCs were already converted into active replisomes for both genotypes, as all three replication factors, CDC6, CDC45 and GINS2, were bound to the chromatin. However, by 24 h, the amount of chromatin-bound CDC45 and GINS2 in *Cdk1*^*AF*^ fibroblasts was reduced compared with controls. Similarly, the recruitment of proliferating cell nuclear antigen (PCNA), a DNA clamp that stabilizes active replisomes on chromatin and facilitates leading strand synthesis during DNA replication [59, 60], was also reduced in mutant fibroblasts compared with control cells. As shown in Figure S1C, the amount of soluble CDC6 was comparable for both genotypes at any given time, while CDC45, GINS2 and PCNA levels were reduced in mutant cells. Moreover, by 30 h, hardly any replication factors were detected on chromatin in *Cdk1*^*AF*^ MEFs (Fig. 2e). Overall, our data indicate that mutant fibroblasts assemble pre-RC and initiate DNA synthesis rather normally. However, impaired DNA replication in *Cdk1*^*AF*^ MEFs is likely associated with reduced numbers of active replisomes, which could contribute to the S phase arrest. The lack of replication factors bound to chromatin at 30 h in mutant cells might suggest failure of surveillance mechanisms to stabilize replication forks, which results in DNA breakage [61].

To understand the molecular basis underlying the interrupted S phase, we evaluated the expression levels and the associated kinase activity of key cell cycle regulators including CDK1, CDK2, cyclin B1 and cyclin A2 at different time points after release (Fig. 2f, g). CDK1/cyclin B1 complexes are known to be the main regulators of mitosis [62, 63], whereas CDK2 in complex with cyclin A2 controls S phase progression [64–66]. At the G_1_/S transition (14 h), the protein expression profiles of control and *Cdk1*^*AF*^ MEFs were similar, with only a mild reduction in CDK1 and cyclin A2 levels in mutant cells (Fig. 2f). As the cells progressed into S phase, these differences in protein expression levels between control and mutant cells became more pronounced. In control cells, CDK1 was expressed as early as 14 h after serum starvation and the protein levels gradually increased until it reached a maximum around 24–30 h. Moreover, only in control MEFs we detected the shift in electrophoretic mobility for CDK1 (Fig. 2f), which is likely related to its phosphorylation status [8, 19]. To verify whether the molecular shift was associated with inhibitory phosphorylation of CDK1 on Y15, we performed western blotting using antibodies against phospho-Y15 (pY15). In control MEFs, pY15 was detected between 18 and 30 h when most of the cells progress from S phase to mitosis. In *Cdk1*^*AF*^ MEFs, no pY15 was detected, which indicates the lack of WEE1-mediated phosphorylation in both CDK1 and CDK2 (the pY15 antibody recognizes both). In addition, cyclin B1 and A2 were expressed at much lower levels in mutant cells compared with controls (Fig. 2f).

To investigate the kinase activities associated with CDK/cyclin complexes, we performed in vitro kinase assays. During early S phase (16 h), control MEFs exhibited low kinase activities associated with all tested CDKs and cyclins (Fig. 2g), as expected. The kinase activities increased gradually with a peak around 21 h for CDK2/cyclin A2 and at 30 h for CDK1/cyclin B1 complexes. In contrast, the kinase activity associated with CDK1 and cyclin B1 was elevated in *Cdk1*^*AF*^ MEFs compared with controls. These were detected as early as 16 h and peaked at 21 h despite that cyclin B1 levels were low (Fig. 2g). This premature activity of CDK1^AF^ was expected because in the absence of WEE1 phosphorylation, CDK1 is activated immediately by binding to the cyclin subunit (assuming that CDK1 is fully phosphorylated on T161 by CDK-activating kinase [CAK] as has been shown before [67]). Furthermore, *Cdk1*^*AF*^ MEFs exhibited a significant decrease in kinase activities associated with CDK2 and cyclin A2 compared with controls (Fig. 2g).

### Premature mitotic events are observed in S phase in *Cdk1*^*AF*^ MEFs

**Fig. 3:**
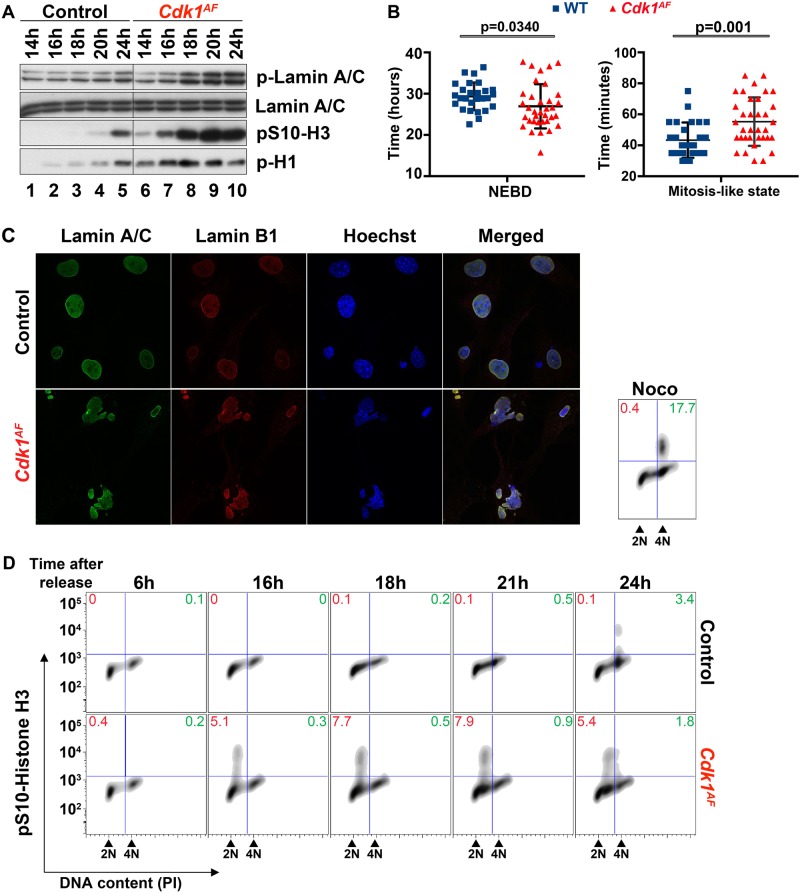
Premature mitotic events in *Cdk1*^*AF*^ MEFs. MEFs were synchronized at G_0_/G_1_ as described in Materials and methods section. **a** Cells were harvested at different times after release from serum starvation. Protein extracts from *Cdk1*^*+/SAF*^ and *Cdk1*^*+/AF*^ MEFs were subjected to western blotting using the indicated antibodies. Lamin A/C served as a loading control. **b** By using live cell imaging in *Cdk1*^*+/SAF*^ (*n* = 27) and *Cdk1*^*+/AF*^ (*n* = 35) MEFs, NEBD was monitored by the disappearance of GFP-lamin A/C expression, while mitosis-like state was calculated by the timing between 53BP1 re-accumulation and NEBD (see Figure S2A). Statistical significance was assessed by unpaired *t-*test with Welch’s correction. **c** *Cdk1*^*+/SAF*^ and *Cdk1*^*+/AF*^ MEFs were fixed 24 h after release from serum starvation. The integrity of nuclear envelope was determined by immunofluorescence staining with antibodies against lamin A/C and Lamin B1. DNA was stained with Hoechst dye. **d** DNA condensation was determined in *Cdk1*^*flox/SAF*^ and *Cdk1*^*null/AF*^ MEFs at different time points after serum starvation by pS10-histone H3 FACS. DNA was stained with PI. Red font – premature DNA condensation in S phase; green font – DNA condensation at G_2_/M; Noco – nocodazole. Nocodazole-treated *Cdk1*^*flox/SAF*^ cells served as a positive control. Results are representative of three independent experiments

The conserved mechanism leading to mitotic entry is controlled by the WEE1/MYT1 – CDC25 regulatory loop, which regulates CDK1 activity by determining the phosphorylation status of T14 and Y15 [5, 6, 10–12, 14–17, 68]. At the end of G_2_ phase, the CDC25 phosphatase dephosphorylates CDK1 leading to its immediate activation. Upon CDK1/cyclin A2 activation, the nuclear envelope (NE) breaks down and CDK1/cyclin B1 complexes accumulate in the nucleus causing a sequence of events [69]. CDK1 phosphorylates the nuclear lamina leading to nuclear envelope break down (NEBD) [70, 71]. CDK1 also triggers chromosome condensation due to histone modification including phosphorylation of histone H3 on S10 by Aurora kinase B [72, 73], and induces the phosphorylation of histone H1, which is one of the first known indicators of mitosis [74]. To investigate whether the increase in CDK1 activity during S phase leads to premature mitotic events in *Cdk1*^*AF*^ MEFs, we determined the expression of mitotic markers in *Cdk1*^*AF*^ MEFs. CDK1-dependent phosphorylation of Lamin A/C on S22 and S392 inhibits the assembly of nuclear lamina and is associated with NEBD [75, 76]. As shown in Fig. 3a, control MEFs displayed elevated phosphorylation of Lamin A/C on S22 only 24 h after release, at a time when cells start to enter mitosis. Interestingly, the increase in phosphorylation on S22 in mutant MEFs was already detected at 18 h when cells were still in S phase and remained at high levels until 24 h. To further investigate the nuclear lamina disassembly in *Cdk1*^*AF*^ MEFs, we monitored the cell cycle progression of mutant cells via time-lapse microscopy using stable expression of green fluorescent protein (GFP)-Lamin A/C and 53BP1-mCherry [77, 78]. The GFP-Lamin A/C construct was used to directly monitor NE disassembly, whereas 53BP1-mCherry helped to visualize the accumulation of DNA damage and NE reassembly [79–81]. We determined the mitosis-like state as a time range between NEBD and NE reassembly. NEBD occurred in control MEFs approximately 2.5 h later compared with *Cdk1*^*AF*^ MEFs (Fig. 3b, left panel, S2A). Interestingly, *Cdk1*^*AF*^ MEFs remained longer in mitotic-like state (55 min) compared with control fibroblasts (43 min) (Fig. 3b, right panel). Moreover, *Cdk1*^*AF*^ MEFs were unable to properly reassemble nuclear lamina (Figure S2B) and exited the mitotic-like state with distorted nuclear architecture (Fig. 3c, S2B).

To further characterize the mitotic phenotype of *Cdk1*^*AF*^ MEFs, we determined the presence of mitotic markers including posttranslational modifications of histones H1 and H3. It has been shown that phosphorylation of histone H1 occurs early in the cell cycle and reaches its saturation in mitosis [74]. In line with this study, low levels of phosphorylation of histone H1 in control MEFs were detected when cells entered S phase (16 h) and then gradually increased until 24 h (Fig. 3a, lanes 2–5). In contrast, high levels of phospho-histone H1 persisted in *Cdk1*^*AF*^ MEFs through the time course of the experiment. As shown in Fig. 3a, the phosphorylation of histone H3 on S10 in control cells was detected at 24 h after release, which overlapped with increased phosphorylation of Lamin A/C on S22 and histone H1, suggesting entry into mitosis at that time. On the other hand, *Cdk1*^*AF*^ MEFs exhibited premature and elevated phosphorylation of histone H3 on S10 as indicated by western blot (Fig. 3a) and FACS (Fig. 3d) at 16 h after release. Importantly, FACS analysis revealed that premature phosphorylation of histone H3 on S10 occurred in cells with 2 n DNA content, suggesting that an early condensation of incompletely replicated DNA might trigger further complications during cell division.

### Interrupted S phase in *Cdk1*^*AF*^ MEFs causes chromosomal fragmentation and intra-S phase checkpoint activation

**Fig. 4:**
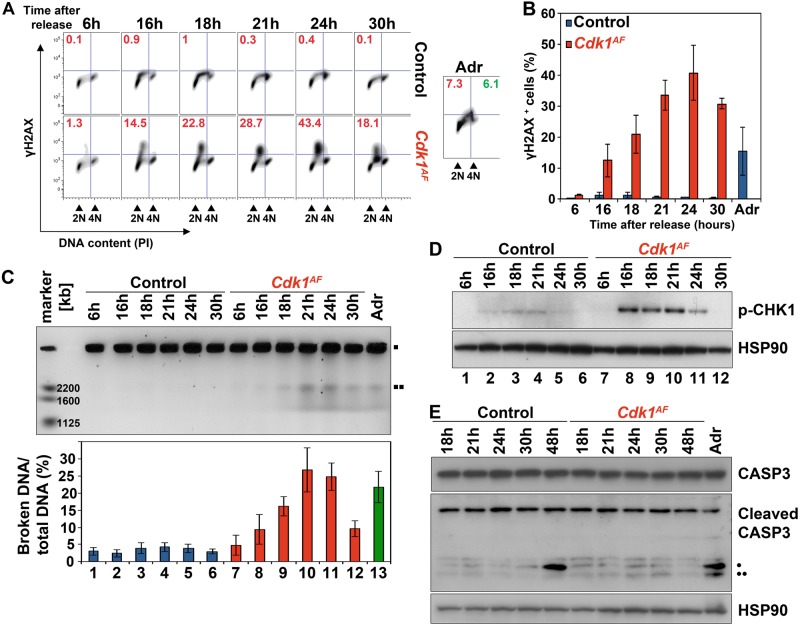
DDR in *Cdk1*^*AF*^ MEFs. MEFs were prepared as described in Materials and methods section. **a** The level of DNA damage was measured in synchronized *Cdk1*^*flox/SAF*^ and *Cdk1*^*null/AF*^ MEFs by phospho-H2AX FACS. DNA content was determined by PI staining. Red font – DNA damage in S phase; green font – DNA damage at G_2_/M. Adriamycin-treated (Adr) *Cdk1*^*flox/SAF*^ cells served as a positive control. **b** Quantitative analysis of DNA damage from (**a**). Data are represented as mean ± SD from at least two independent experiments. **c** Chromosomal fragmentation was assessed in *Cdk1*^*flox/SAF*^ and *Cdk1*^*null/AF*^ MEFs by PFGE. The amounts of broken DNA were normalized to intact DNA and control at 6 h using ImageJ.  intact DNA;  DNA breaks. **d** Western blots for phosphorylated CHK1 in control and *Cdk1*^*AF*^ MEFs. Hsp90 served as a loading control. **e** Western blots for total (35 kDa) and cleaved CASP3 (• 19 kDa; •• 17 kDa bands) and HSP90 in *Cdk1*^*flox/SAF*^ and *Cdk1*^*null/AF*^ MEFs at the indicated time points after release from serum starvation. Adriamycin-treated (Adr) *Cdk1*^*flox/SAF*^ MEFs served as a positive control

So far, our data indicate that CDK1^AF^ expression leads to interruption of DNA replication in MEFs while it causes a prominent increase of γH2AX staining in tissues, indicating DNA damage. In order to elucidate the underlying molecular mechanisms responsible for the observed phenotype, we verified the presence of DNA damage in MEFs by FACS analysis using antibodies against γH2AX (Fig. 4a). γH2AX in control MEFs was barely detectable and never exceeded 1% of the total signal, likely caused by physiological DNA lesions during S phase. In contrast, *Cdk1*^*AF*^ MEFs displayed substantial levels of γH2AX as soon as cells entered S phase (Fig. 4b; 12 ± 5% at 16 h) and reached its maximum after 24 h (41 ± 9%). *Cdk1*^*AF*^ cells had higher γH2AX levels than control cells treated with Adriamycin, a DNA-damaging agent (15 ± 8% of cells). Importantly, γH2AX-positive cells were mostly restricted to cells with under-replicated DNA (≈3 n DNA content), which may suggest that mutant fibroblasts underwent replication stress.

To confirm whether CDK1^AF^-induced replication stress is associated with chromosomal fragmentation, we analyzed the formation of DNA breaks by pulsed field gel electrophoresis (PFGE) (Fig. 4c). We could not detect any DNA fragmentation in control MEFs. In contrast, *Cdk1*^*AF*^ MEFs exhibited a gradual increase in chromosomal breakage with a peak around 21–24 h (lanes 8–11), which was higher than the amount of DNA breaks in Adriamycin-treated fibroblasts (Fig. 4c, lane 13).

To further decipher whether DNA damage induced by CDK1^AF^ activates DDR, we examined the phosphorylation status of the DDR regulator CHK1. In control MEFs, phosphorylated CHK1 was barely detectable (Fig. 4d, lanes 1–6). In contrast, *Cdk1*^*AF*^ MEFs displayed significant levels of CHK1 phosphorylated on S345 at 16–24 h compared with control cells (Fig. 4d, lanes 7–12), similar as has been reported for intra-S phase DNA damage induced cells [82].

Chromatin condensation and DNA fragmentation can be triggered by caspase-3, a well-known hallmark of programmed cell death [83], which undergoes autocatalytic cleavage to become fully active [84, 85]. To determine whether the observed DNA breaks in mutant cells are the result of replication stress or apoptosis, we evaluated the expression levels of cleaved caspase-3 at different time points after serum starvation release (Fig. 4e). We detected significant levels of cleaved caspase-3 in Adriamycin-treated (Adr) control cells (19 kDa and 17 kDa bands), as well as in control MEFs at 48 h (19 kDa band). Nevertheless, we did not detect cleaved caspase-3 in mutant cells at any of analyzed time points. Taken together, our data indicate that apoptosis is most likely the long-term consequence of DNA damage triggered by prematurely active CDK1.

### Depletion of *Mus81* partially rescues DNA damage and S phase arrest in *Cdk1*^*AF*^ cells

Controlled processing of joint molecule intermediates of homologous recombination (HR), which often consists of four-way structures known as Holiday Junctions (HJ), is indispensable for proper chromosome segregation in mitosis [86]. The BLM–TopoIIIα–RMI1–RMI2 (BTR complex) is known to dissolve HJs, whereas MUS81–EME1–SLX1–SLX4 and GEN1 endonuclease complexes contribute to the resolution of recombination intermediates [87]. Proliferating cells preferentially use BTR in S phase [88], whereas MUS81–EME1–SLX1–SLX4 and GEN1 cleave DNA at G_2_/M phase and mitosis, respectively [89]. Importantly, GEN1 functions are restrained by nuclear exclusion [86, 90], whereas MUS81 activation relies on CDK1 activity [91, 92]. CDK1 phosphorylates both MUS81 and its scaffold protein, SLX4, promoting an assembly of active MUS81–SLX4 complex on chromatin [91, 92]. Therefore, we aimed to investigate the status of the MUS81–SLX4 complexes in *Cdk1*^*AF*^ MEFs.

**Fig. 5:**
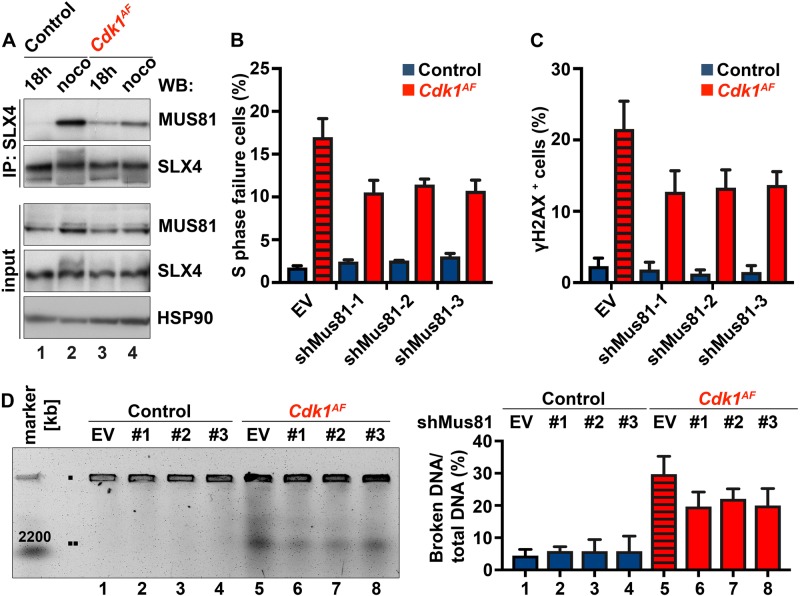
*Mus81* knockdown partially rescues CDK1^AF^-induced defects in MEFs. **a** MEFs were prepared as described in Materials and methods section. *Cdk1*^*flox/SAF*^ and *Cdk1*^*null/AF*^ cells were harvested in S phase (18 h) or mitosis/mitotic-like state (noco). Noco – nocodazole. *Cdk1*^*flox/SAF*^ MEFs were treated with nocodazole from 21 to 26 h to arrest them at metaphase–anaphase transition, while the drug was added to *Cdk1*^*null/AF*^ MEFs from 16 to 21 h to capture cells in mitotic-like state. Protein extracts from *Cdk1*^*flox/SAF*^ and *Cdk1*^*null/AF*^ MEFs were subjected to immunoprecipitation with antibodies against SLX4 (IP: SLX4) followed by immunoblotting using the indicated antibodies. The endogenous levels of MUS81 and SLX4 were verified by western blotting (input). HSP90 served as a loading control. Results are representative of three independent experiments. **b** S phase failure and **c** the levels of DNA damage were determined by FACS as previously described (Figs. 2b and 4a, respectively). Striped red column indicates *Cdk1*^*AF*^ MEFs infected with an empty vector. Quantitative analyses of FACS data are shown. Results are represented as mean ± SD. **d** Chromosomal fragmentation was analyzed by PFGE as described in Fig. 4c. Results (**b**–**d**) are representative of three independent experiments

In control cells, we were able to detect SLX4-bound MUS81 complexes in mitotic extracts derived from cells arrested by nocodazole (noco) (Fig. 5a, lane 2) but not in S phase (18 h) (Fig. 5a, lane 1), which was expected because CDK1 activity peaks in mitosis and phosphorylates MUS81 and SLX4 [91–93]. However, in *Cdk1*^*AF*^ MEFs, MUS81–SLX4 complexes were already present in S phase (Fig. 5a, lane 3). The treatment of mutant cells with nocodazole (Fig. 5a, lane 4) only minimally increased the amount of SLX4-bound MUS81 compared with 18-h time point because *Cdk1*^*AF*^ cells do not enter mitosis. Collectively, these data indicate premature formation and activation of MUS81–SLX4 complexes in *Cdk1*^*AF*^ MEFs during S phase.

To further examine whether prematurely assembled MUS81–SLX4 complexes are responsible for chromosomal fragmentation in mutant cells, we silenced *Mus81* in mutant MEFs. To verify the depletion of *Mus81* in control cells after retroviral infection, we used three different short hairpin RNAs (shRNAs) directed against the *Mus81* transcript. All infected cells displayed reduced amounts of *Mus81* mRNA (Figure S3A-B) and protein (Figure S3C) compared with cells infected with an empty vector. The most efficient knockdown (KD) was achieved in cells treated with shMus81-2. Subsequently, we examined the S phase failure and assessed the levels of DNA damage in *Cdk1*^*AF*^ shMus81 cells by BrdU and γH2AX FACS, respectively. We observed an S phase block in *Cdk1*^*AF*^ cells (Fig. 5b, S3D; empty vector) with higher percentage of cells positive for γH2AX (Fig. 5c; empty vector). Silencing of *Mus81* in *Cdk1*^*AF*^-expressing cells, using any of the tested shRNA against Mus81, reduced the number of cells with under-replicated DNA by around 35% compared with mutant cells infected with an empty vector (Fig. 5b). We observed a similar reduction in γH2AX-positive cells upon *Mus81* silencing (Fig. 5c) associated with a lower amount of detected DNA fragmentation by PFGE in *Cdk1*^*AF*^ MEFs (Fig. 5d). Taken together, our data suggest that the premature formation of MUS81–SLX4 endonuclease complexes partially contributes to DNA fragmentation during S phase in *Cdk1*^*AF*^ MEFs.

### p53 status contributes to WEE1 inhibitor sensitivity

**Fig. 6:**
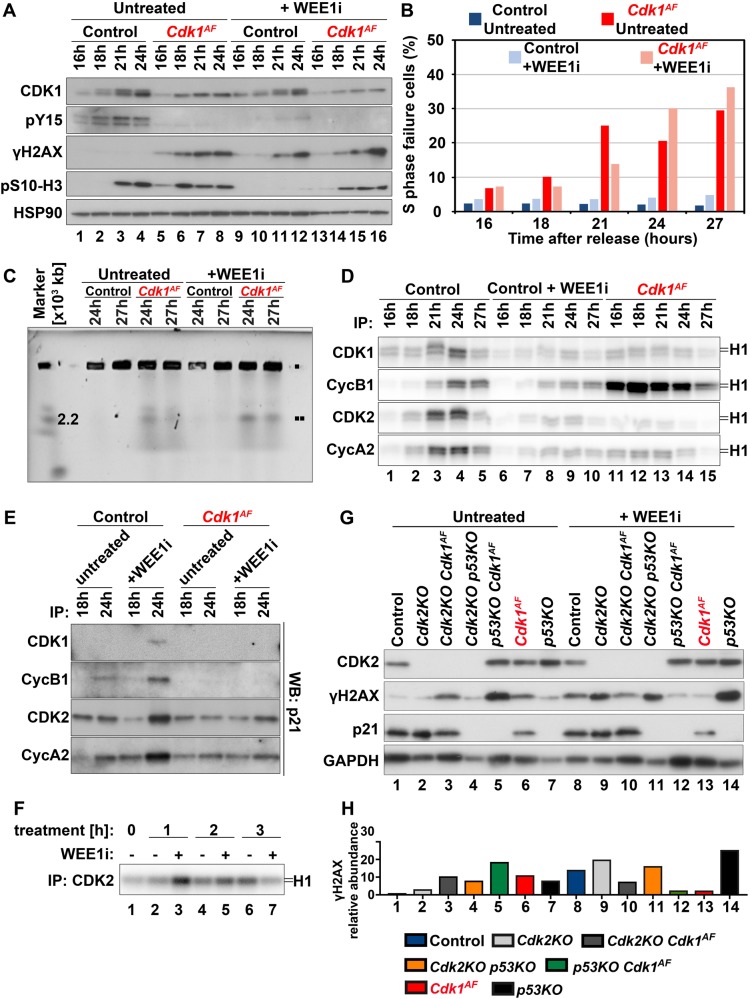
Different surveillance control mechanisms in WEE1-inhibited and *Cdk1*^*AF*^ cells. Primary MEFs were synchronized at G_0_/G_1_ by serum starvation and then released into full serum medium in the presence or absence of WEE1i (1 μM). **a** Protein extracts from control (*Cdk1*^*flox/SAF*^) and *Cdk1*^*AF*^ (*Cdk1*^*null/AF*^) MEFs treated with/without WEE1i were subjected to western blotting using the indicated antibodies. HSP90 served as a loading control. **b** S phase failure was determined by BrdU FACS, whereas in **c** DNA fragmentation was visualized by PFGE in all four experimental groups. **d** Protein extracts from (**c**) were subjected to immunoprecipitation with the indicated antibodies. Kinase activities of the immunoprecipitates were determined by in vitro kinase assays as previously described. **e** The abundance of p21 bound to different CDKs and cyclins was determined in protein lysates by co-immunoprecipitation with the indicated antibodies followed by western blotting against p21. **f** WEE1i was added to control MEFs at 21 h after release from serum starvation (time point 0). Cells were collected at the indicated time points (1, 2 and 3 h) in the presence or absence of WEE1i. Protein extracts were isolated and subjected to immunoprecipitation with CDK2 antibodies. Kinase activities associated with CDK2 were measured by in vitro kinase assays. **g** Protein extracts at the 27-hour time point from control (*Cdk1*^*flox/SAF*^), *Cdk2KO*, *Cdk2KO Cdk1*^*AF*^ (*Cdk2KO Cdk1*^*null/AF*^), *Cdk2KO p53KO*, *p53KO Cdk1*^*AF*^ (*p53KO Cdk1*^*null/AF*^), *Cdk1*^*AF*^ (*Cdk1*^*null/AF*^) and *p53KO* cells. MEFs treated with/without WEE1i were subjected to western blotting using the indicated antibodies. GAPDH served as a loading control. **h** Relative abundance of γH2AX and in samples presented in panel (**g**) upon previous normalization to GAPDH

So far, we have demonstrated that the loss of inhibitory phosphorylation on CDK1 results in its premature activation and promotes MUS81-dependent DNA damage in S phase. Previous work established that early activation of CDK1 could be achieved by the inhibition of its negative regulator, the WEE1 kinase [91]. However, WEE1 disruption through silencing or drug inhibition affects the regulation of both CDK1 and CDK2 [35] whereas our genetic model prevents only inhibitory phosphorylation of CDK1 on T14 and Y15. Thus, we investigated whether the genetic ablation of both such sites on CDK1 differs from pharmacological inhibition of the WEE1 kinase. To compare the effects of WEE1 inhibition with CDK1^AF^ expression, we treated control and mutant MEFs with the small-molecule WEE1 inhibitor, MK-1775 (hereafter referred to as WEE1i) [94]. Cells were synchronized at G_0_/G_1_ by serum starvation and released into 10% serum medium in the presence of WEE1i. First, we tested the expression levels of CDK1 and Y15 at different time points after release (Fig. 6a). As expected, in control cells CDK1 expression levels gradually increased until reaching a maximum at 24 h. Interestingly, only in control cells, the shift in electrophoretic mobility for CDK1 was detected (Fig. 6a, lanes 1–4). Faint Cdk1 phosphorylation mobility shift in control cells treated with WEE1i (lanes 9–12) might have been due to T161 phosphorylation [8, 9]. Importantly, Y15 phosphorylation remained undetectable in any of analyzed cells but control MEFs (lanes 1–4), which confirms the efficiency of WEE1i in blocking Y15 phosphorylation under these conditions (lanes 9–12). We further assessed the contribution of WEE1i to DNA damage by immunoblotting against γH2AX (Fig. 6a). As expected, γH2AX was only detected in cells either harboring *Cdk1*^*AF*^ mutation (lanes 5–9, 13–16) or those treated with WEE1i [9–16]. Interestingly though, *Cdk1*^*AF*^ cells treated with the inhibitor (lanes 9–12) displayed DNA damage at later time points compared with untreated mutant MEFs (lanes 5–8). The possible delay in cell cycle progression was likely due to WEE1 inhibition.

To address whether WEE1 inhibition affects the timing of mitotic entry, we examined phosphorylation of histone H3 on S10. As we have shown before in Fig. 3a, phosphorylation of histone H3 on S10 occurs prematurely in *Cdk1*^*AF*^ MEFs compared with control (Fig. 6a, lanes 5–8 vs 1–4). Intriguingly, in control WEE1i-treated cells phospho-S10 signal remained undetectable (lanes 9–12), indicating that WEE1i-treated wild-type MEFs did not enter mitosis at the analyzed time points. Again, the delay in S10 phosphorylation in WEE1i-treated mutant cells (lanes 13–16), in comparison with untreated *Cdk1*^*AF*^ MEFs (lanes 5–8), possibly results from slower progression into mitotic-like state in the presence of the inhibitor.

To test whether WEE1-inhibited control cells arrest in S phase, as observed in *Cdk1*^*AF*^ MEFs (see Figs. 2b, c), we performed FACS analysis and noted that cells with ≈3 n DNA content (BrdU-negative population with under-replicated DNA) characteristic for *Cdk1*^*AF*^ cells, were not observed as a result of WEE1 inhibition in control MEFs during the first 27 h (Fig. 6b, S4A). This unexpected discovery suggests that although both WEE1 inhibition and CDK1^AF^ expression lead to DNA damage, the underlying molecular mechanisms could be distinct. Next, we examined whether DNA breaks are formed in control cells treated with WEE1i using PFGE analysis. We did not detect DNA breakage in control cells treated with WEE1i at the indicated time points, whereas DNA fragmentation was observed in *Cdk1*^*AF*^ cells with or without WEE1i treatment (Fig. 6c).

In addition, we examined kinase activities associated with different CDK/cyclin complexes. We carried out in vitro kinase assays after immunoprecipitation in untreated control and *Cdk1*^*AF*^ MEFs, as well as control cells treated with WEE1i (Fig. 6d). Untreated control MEFs exhibited low kinase activities at 16-h time point but increased gradually as cells were progressing through S toward G_2_/M phase (lanes 1–5). Surprisingly, kinase activities in cells treated with WEE1i was barely detectable at 16 and 18 h after release, which might indicate slower replication progression through S phase (lanes 6–10). As expected, *Cdk1*^*AF*^ cells displayed elevated kinase activity associated with CDK1 and cyclin B1 (lanes 11–15) compared with untreated control cells (lanes 1–5).

The differences in CDK1 activity between untreated *Cdk1*^*AF*^ and WEE1i-treated control MEFs were unexpected, hence we aimed to further investigate this phenotype. The expression levels of p21^Cip1/Waf1^, a universal inhibitor of multiple CDK/cyclin complexes [95, 96] and a component of the DNA damage checkpoint [97, 98], was similar in untreated control and *Cdk1*^*AF*^ MEFs (Figure S4C, lanes 1–5, 11–15). In contrast, in MEFs treated with WEE1i, p21 levels gradually increased from 21-h time point (Figure S4C, lanes 6–10). Next, we determined the abundance of p21 bound to different CDKs and cyclins. At 18-h time point, the levels of p21 bound to CDK1, CDK2, cyclin A2 and cyclin B1 were comparable in control and *Cdk1*^*AF*^ MEFs with and without WEE1i treatment (Fig. 6e). Intriguingly though, at the 24-h time point we found elevated p21 bound to all aforementioned CDKs and cyclins in WEE1i-treated control cells (Fig. 6e), which could explain the low kinase activities (see Fig. 6d, lanes 6–10). To investigate the possible impact of CDK2 inhibition in control MEFs treated with WEE1i, at 21 h after serum release, we added WEE1i for 1, 2 or 3 h (Fig. 6f). As expected, at 1 h we observed low CDK2 activity in untreated cells (lane 2), which significantly increased after WEE1i treatment (lane 3). After 2 h WEE1i treatment, CDK2 activity was only slightly elevated in MEFs compared with their untreated equivalents (lanes 4–5), while after 3 h WEE1i treatment, CDK2 activity was lower than in the untreated cells (lanes 6–7). Therefore, CDK2 kinase activity is only transiently induced by WEE1i, followed by its immediate inhibition presumably due to induction of DNA damage and p21 binding.

WEE1i-induced cellular sensitivity in primary MEFs partially differs from its response in cancer cell lines [34, 41, 91]. One of the explanation of the distinct results in previously published reports is the variable p53 status, which is known to affect cell cycle arrest, DNA repair, as well as apoptosis [99]. To address whether p53 deficiency exacerbates DNA damage in cells with prematurely active CDK1, we compared expression levels of γH2AX and p21 in *p53KO*, *Cdk1*^*AF*^ MEFs, and *p53KO Cdk1*^AF^ MEFs, with and without WEE1i treatment. Interestingly, *p53KO* MEFs exhibit increased γH2AX phosphorylation (Figs. 6g, h, S4F), which likely was due to the inhibited p21 expression (Figs. 6g, S4D-E). Intriguingly, p53 depletion in *Cdk1*^*AF*^ cells aggravate DNA damage when compared with *Cdk1*^*AF*^ and *p53KO* single mutants (Fig. 6g, h) but was reduced by 7.6-fold when double mutant cells were treated with WEE1i (Figs. 6g, h, S4D). The reduction in DNA damage in *p53KO Cdk1*^*AF*^ cells could be the consequence of the WEE1i off-target effects.

It has been shown that CDK2 is dispensable for tumorigenesis induced by the loss of p53 in mice [100] but its depletion rescues the DNA damage induced by premature activation of CDK1 due to WEE1 inhibition in breast and ovarian cancer cell lines [35]. In our study, we have presented that the kinase activity associated with CDK2/cyclin A2 complexes is downregulated in *Cdk1*^*AF*^ MEFs (see Figs. 2g and 6d). To address whether residual activity of CDK2 could contribute to observed cell cycle defects induced by CDK1^AF^, we monitored the consequences of CDK2 depletion in *Cdk1*^*AF*^ MEFs, with and without WEE1i treatment. Importantly, the *Cdk1*^*AF*^ phenotype did not depend on CDK2 but the addition of WEE1i reduced DNA damage in *Cdk2KO Cdk1*^*AF*^ (Fig. 6g, h). Interestingly, in comparison with the *p53KO* single mutant, CDK2 deficiency also contributed do γH2AX reduction in *Cdk2KO p53KO* cells (Figs. 6g, h, S4D). Taken together, *Cdk1*^*AF*^ and WEE1i-treated control MEFs seem to bear partially distinct cell cycle control mechanisms, which rely on the p53 status that sensitizes cells to WEE1i.

### The CDK1^AF^ expression inhibits liver tumorigenesis

**Fig. 7:**
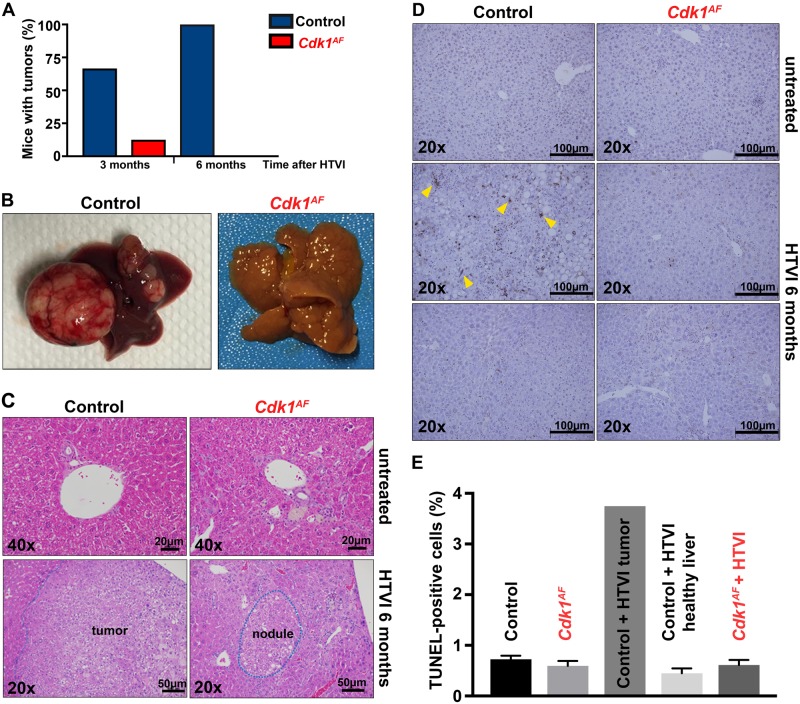
CDK1^AF^ expression inhibits liver tumorigenesis. Eight weeks old *Cdk1*^*+/+*^ (control) and *Cdk1*^*+/SAF*^ Alb-CreER^T2^ (*Cdk1*^*+/AF*^ after injection) mice were IP injected with tamoxifen to induce *Cdk1*^*+/AF*^ expression in hepatocytes. Two to three weeks later, tamoxifen-treated mice were subjected to hydrodynamic tail vein injection (HTVI) with tumorigenic mix of activated Ras and shRNA against p53 to initiate liver tumorigenesis. **a** Quantitative analysis of tumor formation 3 (control *n* = 6, *Cdk1*^*+/AF*^ *n* = 8) and 6 months (control *n* = 4, *Cdk1*^*+/AF*^ *n* = 4) after HTVI are presented. **b** Six months after HTVI, mice (four animals per experimental group) were sacrificed and livers/liver tumors were harvested. **c** Hematoxylin and eosin (H&E) staining of liver sections from *Cdk1*^*+/+*^ and *Cdk1*^*+/AF*^ mice. **d** TUNEL assay representative images from untreated and subjected to HTVI control and *Cdk1*^*+/AF*^ mice (two animals per experimental group). Yellow arrows indicate apoptotic cells. **e** Quantitative analysis of TUNEL assay presented in panel (**d**)

Our findings indicate that the CDK1^AF^-induced defects are most likely restricted to proliferating cells. Previously, we had shown that CDK1 is essential for cell division and its depletion inhibits liver tumorigenesis [2]. To further investigate the consequences of *Cdk1*^*AF*^ mutation in vivo, we tested whether the expression of CDK1^AF^ would affect liver tumorigenesis. We examined whether liver-specific *Cdk1*^*AF*^ mice were able to develop liver tumors by injecting them with tumorigenic mixture of activated oncogene Ras and shRNA against p53, via hydrodynamic tail vein injection (HTVI) technique [2, 101, 102]. Liver tumors were detected in two-thirds of control mice and only in one *Cdk1*^*AF*^ mouse at 3 months after HTVI (Fig. 7a). The latter was most likely due to the loss of CDK1^AF^ expression as confirmed by PCR genotyping (data not shown). By 6 months, all control animals developed liver tumors, which differed in size (Figs. 7a, b). *Cdk1*^*AF*^ mice did not develop macroscopic liver tumors up to 6 months after induction, at which point all animals were sacrificed. On the microscopic level, in contrast to untreated control livers, samples from *Cdk1*^*AF*^ animals displayed signs of liver damage, such as inflammation and fibrosis (Fig. 7c). Hepatocytes, uniform in shape and size, and organized in hepatic plates in healthy control liver, displayed variations in sizes and aberrant nuclear morphology, with small, big or irregular nuclei in the *Cdk1*^*AF*^ liver. Liver tumors, encompassing steatotic tumor cells and confined within the boundaries of tumor microenvironment, were detected in control but not in mutant livers. Instead, *Cdk1*^*AF*^ mice displayed focal nodular hyperplasia surrounded by fibrous tissues. The altered hyperplastic hepatocytes looked rather normal and seemingly arranged in cords. Hence, liver tumor development was inhibited in *Cdk1*^*AF*^ mice, suggesting that the expression of the mutant protein did not allow cancer cells to expand.

We have shown that *Cdk1*^*AF*^ blastocysts and *Cdk1*^*AF*^ MEFs were not able to proliferate (see Figs. 1a and 2a, respectively). In spleens, 4 days after tamoxifen injection in Rosa-cre *Cdk1*^*AF*^-expressing mice, we detected increased DNA damage (see Fig. 1b, d) and apoptosis (see Fig. 1e, f). Therefore, we tested whether the observed inhibition of tumor formation in *Cdk1*^*AF*^ livers after 6 months from HTVI is caused by increased apoptosis of malignant cells. The percentage of apoptotic cells in untreated wild-type liver was <1% of counted cells but after 6 months from HTVI administration in tumors developed in treated controls, the number of apoptotic cells increased up to almost 4% (Fig. 7d, e). In contrast, in *Cdk1*^*AF*^ liver the frequency of detected apoptotic cells remained at 1% even after HTVI. Our in vivo data indicate that tumor development was inhibited in *Cdk1*^*AF*^ liver, which suggests that the expression of the mutant protein was toxic for cancer cells. Interestingly though, we did not detect apoptosis in *Cdk1*^*AF*^ liver, which may be due to effective removal of these cells by macrophages or by other means like autophagy, necroptosis, etc.

## Discussion

Using an inducible genetic mouse model, we report that monoallelic expression of CDK1^AF^ in vivo leads to early embryonic lethality in mice (Table 1, Fig. 1a). These data complement our previous study, in which *Cdk1KO* embryos were unable to develop beyond the blastocyst stage [2], indicating that not just the protein expression but also the timely activation of CDK1 is essential during embryogenesis. Importantly, the regulatory phosphorylation of CDK1 is required for survival of adult animals, where it protects genomic integrity by preventing extensive DNA fragmentation (Fig. 1c, d). Our work highlights the importance of stringent T14/Y15 regulation in governing mammalian cell cycle progression in vivo.

To understand the underlying molecular mechanism of CDK1^AF^-induced lethality, we performed our work on MEFs isolated from *Cdk1*^*AF*^ animals. We found that one copy of endogenously expressed CDK1^AF^ (in the presence or absence of a wild-type *Cdk1* allele) is sufficient to trigger premature mitotic events in actively replicating cells leading to replication stress, checkpoint activation, S phase failure and eventually cell cycle arrest (Figs. 2a–c, 4, and S1A). In contrast to previous studies, in which the expression of mutant CDK1 had mild effects on cell cycle progression and mitotic timing in mammalian cell lines [11, 42, 43, 103], *Cdk1*^*AF*^ fibroblasts prematurely enter a mitotic-like state characterized by elevated CDK1 kinase activity, incomplete DNA synthesis, premature chromosome condensation and partially disassembled nuclear lamina (Figs. 2f, 3, and S2). The differences in biological outcomes are likely caused by various expression levels of the mutant protein or depend on the experimental model used. Unlike in endogenously expressed CDK1^AF^, HeLa cells with highly induced CDK1^AF^ expression exhibit cell cycle arrest, whereas cells with moderate levels of mutant CDK1 enter a M-phase-like state prematurely and cycle more rapidly [44]. Moreover, in contrast to our data, survival in CDK1-depleted human cells relies on overexpressed CDK1^AF^ [104]. For aforementioned reasons, in vitro studies in human cell lines with ectopically expressed CDK1^AF^ differ to certain extent from our findings.

The aberrant cell cycle progression of *Cdk1*^*AF*^ MEFs is associated with altered kinase activity of both CDK1/cyclin B1 and CDK2/cyclin A2 complexes (Fig. 2g). Premature activation of CDK1/cyclin B1 in mutant fibroblasts triggers the chromatin recruitment of MUS81–SLX4 complexes during S phase (Fig. 5a). We have shown that chromosomal fragmentation is mediated partly by the MUS81–SLX4 endonuclease complex (Figs. 5b–d and S3D), which is activated in a CDK1-dependent manner [91, 92]. In our work, we present that *Mus81* KD partially rescues DNA break formation (Figs. 5b–d, S3D), which could be due to the compensating action of other endonuclease(s). In human cells, GEN1 like MUS81, can cleave double-stranded replication or recombination intermediates [105]. Therefore, the combined silencing of GEN1 and MUS81 in *Cdk1*^*AF*^ MEFs could potentially inhibit DNA break formation more efficiently.

Our results corroborate the study by Duda et al. [91], where a WEE1 inhibitor was used to block phosphorylation of CDK1 on Y15 to trigger premature activation of the kinase in cancer cell lines. Despite many similarities between these and our findings, the mechanism of WEE1 inhibition and CDK1^AF^ expression do not seem to be exactly the same. In addition to phosphorylation of CDK1 and its role as the gatekeeper of the G_2_/M transition, the WEE1 kinase also regulates inhibitory phosphorylation of CDK2, therefore controlling the onset of DNA replication [106]. The WEE1 kinase plays a crucial role in maintaining genomic integrity through the control of replication fork progression [41]. Unlike silencing WEE1 in human cancer cell lines, where DDR depends on CDK2 but not CDK1 [41], we demonstrate that DNA damage in *Cdk1*^*AF*^ MEFs depends on CDK1 and not on CDK2. First of all, the kinase activity associated with CDK2 is very low in *Cdk1*^*AF*^ MEFs (Fig. 2f) likely due to low levels of cyclin A2 (Fig. 2e). Moreover, we have not seen any significant changes in a phenotype of *Cdk1*^*AF*^ *Cdk2KO* MEFs compared with *Cdk1*^*AF*^ cells (Fig. 6g, h). Second, S phase failure could be rescued by inhibition of CDK1 kinase activity in mutant fibroblasts treated with RO-3306 (Figs. 2d and S1B). Additionally, homozygous *Cdk2*^*AF/AF*^ mice are viable and display only a minimal phenotype [107]. Last but not least, MEFs isolated from those animals exhibit fairly normal cell cycle progression with elevated CDK2/cyclin E kinase activity in the absence of any signs of increased DNA damage.

In our study, we compared WEE1-inhibited control cells with *Cdk1*^*AF*^ MEFs. Our data suggest that the underlying molecular mechanisms of WEE1 inhibition and CDK1^AF^ expression differ in certain aspects. Although WEE1i blocks Y15 phosphorylation of CDK1 and CDK2, it does not induce premature mitotic events, such as DNA condensation or may do so only at a much later time point (Figs. 6a and S4B). Despite detected γH2AX phosphorylation in cells treated with WEE1i (Fig. 6a), we did not observe chromosomal breakage (Fig. 6c), which might be caused by stalled, but not yet broken, replication forks [108]. Indeed, the progression of replication in WEE1i-treated cells is possibly delayed comparing with *Cdk1*^*AF*^ MEFs due to detected elevated levels of p21 bound to CDK2, cyclin A2, as well as CDK1 and cyclin B1 (Fig. 6e). Intriguingly, CDK2 depletion in *Cdk1*^*AF*^ cells does not rescue the mutant phenotype, whereas *p53* deficiency aggravates DNA damage in *Cdk1*^*AF*^ MEFs (Figs. 6g, h, S4C). Our data support previous findings in various cancer cell lines that p53 mutational status sensitizes cells to WEE1i treatment [109, 110] and here we provide a mechanistic explanation because CDK1/cyclin B1 protein levels and activity are increased in *p53KO* [100] exacerbating the DNA damage phenotype.

Both WEE1 and MYT1 kinases are needed to inhibit CDK1 activity. Although WEE1 displays a preference for Tyr15, MYT1 specifically phosphorylates Thr-14. Although MYT1 is believed to be more important in meiosis [111], it still is elusive how it contributes to the somatic cell cycle. Despite that MYT1 depletion does not affect entry into mitosis [112], it is not known whether it compensates the lack of WEE1 in cells treated with WEE1i.

The maintenance of genome integrity is crucial for cell viability and for suppression of neoplastic transformation [113]. The challenges to maintain genome integrity arise during S phase, which is jeopardized by multiple impediments affecting replication fork progression [61]. In the present work, we provide a mechanistic view how prematurely activated CDK1 promotes genome instability by triggering premature mitosis-like events in S phase that negatively affects cellular proliferation. Likely, premature condensation in *Cdk1*^*AF*^ cells creates harmful topological constraints for replication forks and endonucleytic action of MUS81–SLX4 complexes triggers DNA break formation and DNA damage accumulation.

WEE1 is highly expressed in various cancer types, hence is an attractive target for cancer therapy. WEE1i are currently in clinical trials, covering various cancer types. These clinical studies are still at early stage but already suggest that the action of WEE1i may rely on variety of cell type-dependent mechanisms. Thus, future work should focus on accurate determination of WEE1i selectivity and action, especially in combination with DNA-damaging agents, in a range of cancer types.

## Materials and methods

### Mouse strains

*Cdk1*^*AF*^ mice were generated as a conditional knockin strain as previously described [45]. *Cdk1*^*+/AF*^ constitutive expression was obtained by crossing with β-actin-Cre mice [114] (strain name: FVB/N-Tg(ACTB-cre)2Mrt/J; stock no.: 003376; The Jackson Laboratory) or with Rosa26-CreER^T2^ animals [115] to induce CDK1^AF^ expression in adult animals. *Cdk1*^*flox/SAF*^ Rosa26-CreER^T2^ were obtained by mating the latter with conditional *Cdk1*^*flox/flox*^ mice. Primary MEFs were isolated from *Cdk1*^*+/SAF*^ or *Cdk1*^*flox/SAF*^ mice crossed with inducible Esr1-CreER^T2^ strain [116] (strain name: B6.Cg-Tg(CAG-cre/Esr1*)5Amc/J; stock no.: 004682; The Jackson Laboratory). Liver-specific *Cdk1*^*AF*^ knockin was accomplished by crossing *Cdk1*^*+/SAF*^ mice with Alb-Cre [117] and Alb-CreER^T2^ [118] animals. All Cre-mouse lines were backcrossed to C57Bl6 and all mouse lines used in this study were mostly of C57Bl6 background.

Cre-mediated recombination was induced by three consecutive intraperitoneal injections (24-hourly injection of tamoxifen (1 mg of tamoxifen each) Sigma-Aldrich, T5648). Rosa26-CreER^T2^ mice were sacrificed 24 h after the last injection. Mice were housed under controlled environmental conditions, 12 h of light/dark cycle (from 7 a.m. to 7 p.m.), and free access to water and food. All experimental procedures were performed in agreement with Institutional Animal Care and Use Committee guidelines.

*Cdk1*^*+/AF*^ MEFs were infected with the retroviral constructs such as PKB1927 (pBabe-hygro-GFP-lamin A) and PKB1928 (mCherry-human 53BP1-2pLPC-puro), or PKB1167 (pMSCV-∂3′LTR-H2B-GFP) and PKB1928.

### Embryo and blastocyst isolation, culture and synchronization of MEFs

Timed mating between *Cdk1*^*+/SAF*^ and β-actin-Cre mice (for blastocysts) or between *Cdk1*^*flox/SAF*^ Esr1-CreER^T2^ was set up and females were monitored for the signs of mating. Pregnant females were sacrificed to isolate blastocysts after 3 days post coitum or 13.5 days for MEFs isolation. Blastocysts preparation and imaging analysis were performed as previously described [2, 119] after releasing them from the uterine wall by vigorous flushing of phosphate-buffered saline (PBS). Primary MEFs were prepared as previously described [47]. Primary MEFs were cultured under humidified atmosphere with 5% CO_2_ and 3% O_2_, whereas immortalized *p53KO* MEFs were maintained in the presence of 21% O_2_. Cells were synchronized at G_0_/G_1_ by serum starvation (Dulbecco’s modified Eagle’s medium, 0.1% fetal bovine serum and 1% penicillin/streptomycin) for 72 h. The expression of Cdk1^AF^, alone or simultaneously with Cdk1 deletion, was induced by the addition of 4-OHT (40 ng/ml; Sigma-Aldrich, H7904) during the last 48 h of the starvation period. Inhibition of CDK1 was obtained by addition of 10 µM of RO-3306 (Sigma-Aldrich, SML0569) in the medium during the release period.

### Immunohistochemistry

For hematoxylin and eosin (H&E) staining, tissues were fixed in 10% neutral buffered formalin (Sigma-Aldrich, HT501128) for 18–24 h, transferred to ice-cold 70% ethanol and embedded in paraffin blocks followed by the staining of tissues sections.

### Comet assay

Splenocytes were isolated 48 h after the last (third) tamoxifen injection from spleens of adult *Cdk1*^*+/AF*^ Rosa26-CreER^T2^ mice and their littermate controls (tamoxifen-treated *Cdk1*^*+/+*^ Rosa-Cre mice). The spleens were dissected, incubated in PBS-EDTA (20 mM, 5 min), the cell suspension from the supernatant were centrifuged, and adjusted at 4 × 10^5^ cells/ml. Alkaline and Neutral Comet assay were performed on commercial slides accordingly to manufacturer’s protocol (Trevigen, #4250–050-K). After electrophoresis, slides of cells were stained with SYBR Green solution (Maxima SYBR Green qPCR Master Mix (2 × ), Fermentas, K0252) and dried overnight before images acquisition. Images were taken on Zeiss Axioimager Z1 Epifluorescence microscope at ×20 magnification. At least 100 comets were scored in each sample using TriTek CometScore Freeware v1.5 and the amount of DNA breaks was calculated based on the tail moment (Trevigen Instructions, #4250–050-K).

### TUNEL assay

Apoptotic cells were detected by ApopTag Plus Peroxidase In Situ Apoptosis Kit (Millipore #S7101) by labeling and detecting DNA breaks by the indirect TUNEL method.

### Proliferation assay

MEFs were seeded in quintuplicate in 96-well plate at the density of 1500 cells/well. The proliferation was monitored daily for a period of 7 days using the alamarBlue assay (AbD Serotec, BUF012B) and the emitted fluorescence signal (excitation: 560 nm; emission: 590 nm) was measured using a microplate reader SPECTRAmax M2 linked to SoftMax Pro V5 software (Molecular Devices).

### FACS analysis of MEFs

For BrdU incorporation analysis, MEFs were pulse-labeled for 1 h with 100 μM BrdU (BD Pharmingen, 550891) before collection. Collected cells were fixed in –20 °C cold 70% ethanol and washed in blocking buffer (PBS-bovine serum albumin (BSA) 1%; BSA Sigma-Aldrich, A7906). For non-BrdU analysis, cells were permeabilized with PBS-1% BSA-0.5% Triton X-100, whereas, for BrdU analysis, cells were treated with 2 n HCl/0.5% Triton X-100 and neutralized with 0.1 m sodium tetraborate, pH8.5. Cell staining were performed using anti-BrdU (1:500; BD Pharmingen, 555627, clone 3D4), pS10-histone H3 (1:100; Cell Signaling Technology [CST] #9701) or pS139-histone H2AX (1:500; CST #9718) followed by their incubation with secondary antibody Alexa 647-conjugated goat anti-mouse (1:400; Invitrogen, A21235) or Alexa 647-conjugated donkey anti-rabbit (1:400; Invitrogen, A31573). Before analysis, cells were resuspended in PBS supplemented with 2.5 μg/ml propidium iodide (Merck, 537059) and 20 μg/ml RNase A (Sigma-Aldrich, R6513). Analysis of cells and BrdU incorporation was acquired on BD FACSCalibur or BD LSRII flow cytometers (BD Bioscience) and further analyzed using FlowJo 8.8.7 software.

### Protein extraction, immunoblotting, immunoprecipitation and kinase assays

Protein extracts were obtained by lysing cells in CSK/RIPA buffer (see below) or in EBN buffer (80 mM β-glycerophosphate pH7.3, 15 mM MgCl_2_, 20 mM EGTA, 150 mM NaCl, 0.5% NP-40) supplemented with 1 mM DTT, protease inhibitors cocktail (10 μg/ml each of leupeptin, chymostatin, and pepstatin [Chemicon, E18, E16 and E110]) and phosphatase inhibitors (2 mM NaF, Sigma-Aldrich, 71519; 0.1 mM Na_3_VO_4_, Sigma-Aldrich, 450243). Protein concentration was assessed using the BCA assay (Thermo Scientific, 23225). The protein extracts were separated by sodium dodecyl sulfate–polyacrylamide gel electrophoresis (SDS–PAGE) and transferred onto a polyvinylidene difluoride (PVDF) membrane (Millipore, IPVH0010). The membranes were blocked in Tris-buffered saline (TBS) containing 0.1% Tween 20 (TBS-T) and 4% milk (Bio-Rad, 1706404), and subsequently probed overnight at 4 °C with the following primary antibodies: CDK1 (Santa Cruz, sc-954, clone C-19), pY15-CDK1 (CST #9111), CDK1 (1:20,000, rabbit anti-mouse [47]), Cyclin A2 (Santa Cruz, sc-596, clone C-19), cyclin B1 (CST #4135), HSP90 (BD Transduction Laboratories, #610419), MUS81 (University of Dundee, DU 37171), SLX4 (University of Dundee, DU7173), CDC6 (Santa Cruz, sc-9964), CDC45 (CST #3673), GINS2 (Proteintech, #16247-1-AP), PCNA (CST #2586, clone PC10), p-histone H1 (Upstate #12D11), Histone H3 (CST #4499, clone DIH2), p-histone H3 (CST #9701), Lamin A/C (CST #4777), p-Lamin A/C (CST #2026), p-CHK1 (CST, #2348, clone 133D3), p21 (Santa Cruz, sc-6246, clone F-5). Washed membranes were probed with the adequate horseradish peroxidase (HRP)-conjugated secondary antibodies and developed using enhanced chemiluminescence (Immobilon western Chemiluminescent HRP substrate, Millipore, WBKLS0500) and visualized using X-ray film (Fujifilm, 47410 19289).

Affinity purification/immunoprecipitation and kinase assay were carried out as previously described [47]. The antibodies used for immunoprecipitation were either covalently conjugated to Sepharose beads, cyclin A2 (Santa Cruz, sc-751, clone H432), cyclin B1 (Santa Cruz, sc-7393, clone D11) and SUC1 (Millipore, 14132), or pre-coupled to protein A agarose beads (Roche, 11719408001) for CDK1 and CDK2 [47] or SLX4 (University of Dundee, DU 37173). In all, 100–1000 µg of protein extracts was incubated with the respective antibody in EBN buffer.

For kinase assays, immunoprecipitated proteins were incubated in 11 µl of EB buffer supplemented with 10 mM DTT, 15 µM ATP, 5µCi [^ɣ−32^P]-ATP (PerkinElmer, BLU502A) and 1.6 µg of histone H1 (Roche, 11004875001) at room temperature for 30 min, and stopped by addition 5X SDS-sample buffer. Denatured proteins were resolved by SDS–PAGE on 12% gels. The gels were stained with Coomassie Blue and dried. Incorporated radioactivity was determined using a phosphoimager (FLA-7000, Fujifilm).

### Chromatin fractionation and immunoblotting

Chromatin fractionation was performed as previously described [120] with modifications. Collected MEFs were resuspended in CSK buffer (10 mM Hepes-KOH pH7.4, 300 mM sucrose, 100 mM NaCl, 3 mM MgCl_2_, 0.25% Triton-X 100) freshly supplemented with 1 mM phenylmethylsulfonyl fluoride (PMSF), 0.1 mM Na_3_VO_4_ and protease inhibitor cocktail. After incubation (on ice, 5 min) and centrifugation (5000 *g*, 5 min, 4 °C), supernatants containing cytoplasmic and nucleoplasmic soluble proteins were directly snap-frozen in liquid nitrogen, whereas the remaining pellet of chromatin-bound proteins was resuspended and washed twice in CSK buffer. To extract chromatin-bound proteins, pellets were resuspended in RIPA buffer (50 mM Tris-HCl pH7.4, 150 mM NaCl, 1% NP-40, 1 mM EDTA, 0.5% sodium deoxycholate) freshly supplemented with 1 mM PMSF, 0.1 mM Na_3_VO_4_, 0.1 mM NaF and protease inhibitors cocktail.

### Pulsed field gel electrophoresis

Plugs were prepared as previously described [121]. Chromosomes were separated by PFGE (CHEF-DR II System, Bio-Rad), and electrophoresis was performed for 12 h at 6 V/cm with 90 sec pulses, followed by 12 h with 60 sec pulses in 0.5x Tris/Borate/EDTA (TBE) buffer at 14 °C. DNA was visualized by ethidium bromide staining.

### HTVI and liver tumorigenesis

HTVI was performed as previously described [2, 101, 102]. Briefly, 10-week-old mice were injected within 10 s with a cocktail of plasmids [transposase (15 μg pGK-SleepingBeauty13; PKB1094) and transposon (30 μg pT2-Caggs-NRasV12; PKB1095 and 15 μg pT2-shRNAp53; PKB1096] diluted in lactated Ringer’s solution. Animals were injected with a volume corresponding to 10% of their body weight in the lateral tail vein. Animals were monitored for liver tumors and sacrificed 3 and 6 months after the injection unless stated differently.

## Electronic supplementary material

Supplemental Figure legends

Figure S1

Figure S2

Figure S3

Figure S4
