# Correction: Premature activation of Cdk1 leads to mitotic events in S phase and embryonic lethality

**DOI:** 10.1038/s41388-019-1114-x

**Published:** 2019-11-21

**Authors:** Radoslaw Szmyd, Joanna Niska-Blakie, M. Kasim Diril, Patrícia Renck Nunes, Konstantinos Tzelepis, Aurélie Lacroix, Noémi van Hul, Lih-Wen Deng, Joao Matos, Oliver Dreesen, Xavier Bisteau, Philipp Kaldis

**Affiliations:** 1 Institute of Molecular and Cell Biology (IMCB), A*STAR (Agency for Science, Technology and Research), 61 Biopolis Drive, Proteos #3-09, Singapore, 138673 Republic of Singapore; 2 National University of Singapore (NUS), NUS Graduate School for Integrative Sciences and Engineering, Singapore, 117597 Republic of Singapore; 3 Bioinformatics Institute (BII), A*STAR, Singapore, 138671 Republic of Singapore; 4 National University of Singapore (NUS), Department of Biochemistry, Singapore, 117597 Republic of Singapore; 5 ETH Zürich, Department of Biology, Zürich, Switzerland; 6 Institute of Medical Biology, A*STAR, Singapore, 138648 Republic of Singapore; 7 Present Address: Izmir Biomedicine and Genome Center, Dokuz Eylul University Health Campus, 35340 Izmir, Turkey; 8 Present Address: Ecole Polytechnique Federale de Lausanne, School of Life Sciences, CH-1015 Lausanne, Switzerland; 9 Present Address: Wellcome Trust Sanger Institute, Hinxton Cambridge, CB10 1SA UK; 10 Present Address: Department of Chemistry and Centre for Self-Assembled Chemical Structures (CSACS), McGill University, 801 Sherbrooke Street West, Montreal, QC H3A 0B8 Canada

**Keywords:** Cancer models, Mitosis

**Correction to: Oncogene**

10.1038/s41388-018-0464-0

The original version of this article contained an error in the author affiliations.

Affiliation number 7 incorrectly read ‘Izmir Biomedicine and Genome Institute, Dokuz Eylul University, 35340 Izmir, Turkey”. It should be “Izmir Biomedicine and Genome Center, Dokuz Eylul University Health Campus, 35340, Izmir”.

