# Supplementary material for: Premature activation of Cdk1 leads to mitotic events in S phase and embryonic lethality
Source: Oncogene. 2018 Sep 6;38(7):998–1018. doi: 10.1038/s41388-018-0464-0 (PMC6756125; doi:10.1038/s41388-018-0464-0)
Supplement: Supplementary file 1 — Supplemental Figure legends [file 41388_2018_464_MOESM1_ESM.docx]

**Supplemental figure legends**

**Figure S1. Related to Figure 2. Characterization of *Cdk1^AF^* MEFs.** (A) Dot plots depicting BrdU FACS analysis of cell cycle progression of *Cdk1^flox/SAF^* and *Cdk1^null/AF^* MEFs. DNA content was determined by PI staining. (B) Quantification of S phase failure from Figure 2D. Data are represented as mean ± SD from two independent experiments. (C) Protein extracts were isolated from *Cdk1^flox/SAF^* and *Cdk1^null/AF^* MEFs at the indicated time points after serum starvation. The abundance of soluble proteins was examined through cellular fractionation followed by western blotting with the indicated antibodies. HSP90 served as a loading control. Results are representative from two independent experiments.

**Figure S2. Related to Figure 3. Premature mitotic events in *Cdk1^AF^* MEFs.** (A) Lamina reassembly was monitored in *Cdk1^+/SAF^* (n=27) and *Cdk1^+/AF^* (n=35) MEFs expressing mCherry-53BP1, which accumulates in the nucleus while NE is intact, using live cell imaging. Statistical significance was assessed by unpaired t-test with Welch’s correction. (B) NEBD was monitored in *Cdk1^+/AF^* MEFs (n=3) expressing GFP-lamin A/C using live cell imaging. Cells were started being monitored at 8h 15 min after release from serum starvation until 31 hours 50 min (time of recording 23h 35 min).

**Figure S3. Related to Figure 5. *Mus81* knockdown partially rescues CDK1^AF^-induced defects in MEFs.** Following infection with empty vector or shRNA against *Mus81* (shMus81-1/2/3), total RNA was extracted from *Cdk1^+/SAF^* MEFs and used in qRT-PCR to determine Mus81 mRNA levels. Two sets of primers flanking (A) exons 2-4 and (B) 14-16 of *Mus81* transcript were designed. Values were normalized to housekeeping gene (*eEF2*) and to control (empty vector) to derive the fold change using the ∆∆CT method. (C) MEFs were infected with three different shRNA against *Mus81* followed by the selection of positive cells with puromycin. MUS81 expression was examined in *Cdk1^+/SAF^* MEFs infected with empty vector or shRNA against *Mus81* (shMus81-1/2/3) by western blotting. HSP90 was used as a loading control. *** – unspecific band. Cells were synchronized in the G_0_/G_1_ phase by serum starvation for 72 hours. The expression of *Cdk1^+/AF^* was induced upon 4-OHT treatment during the last 48 hours of starvation period. Following serum starvation and 4-OHT induction, cells were released into full serum medium and collected at 24 hours. (D) S phase failure was examined in Cdk1^+/SAF^ and *Cdk1^+/AF^* MEFs at 24 hours after release from serum starvation by BrdU FACS.

**Figure S4. Related to Figure 6. Different surveillance control mechanisms in WEE1-inhibited and *Cdk1*^AF^ cells.** (A) Dot plots depicting BrdU FACS analysis of cell cycle distribution for control (*Cdk1^flox/SAF^*) and *Cdk1^AF^* (*Cdk1^null/AF^*) MEFs in the presence or absence of the WEE1i (1μM). DNA content was determined by PI staining. (B) Phosphorylation of histone H3 on S10 was examined in all four experimental groups at different time points by FACS, as previously described. Quantitative analysis of FACS data is shown. (C) Protein extracts from the following experimental groups (control; control + WEE1i; *Cdk1^AF^*) were subjected to western blotting with the indicated antibodies. HSP90 served as a loading control. (D) Protein extracts at 16, 21, and 27-hour time points from control *Cdk2KO*, *Cdk2KO p53KO,* and *p53KO*. MEFs treated with/without WEE1i were subjected to western blotting using the indicated antibodies. HSP90 served as a loading control. (E) Relative abundance of p21 in samples presented in Figure 6g upon previous normalization to GAPDH. (F) Relative abundance of γH2AX in *Cdk1^AF^* and *p53KO* cells with and without WEE1i treatment (see Figure 6g) normalized to GAPDH.
