# Supplementary figures and images for: Premature activation of Cdk1 leads to mitotic events in S phase and embryonic lethality
Source: Oncogene. 2018 Sep 6;38(7):998–1018. doi: 10.1038/s41388-018-0464-0 (PMC6756125; doi:10.1038/s41388-018-0464-0)

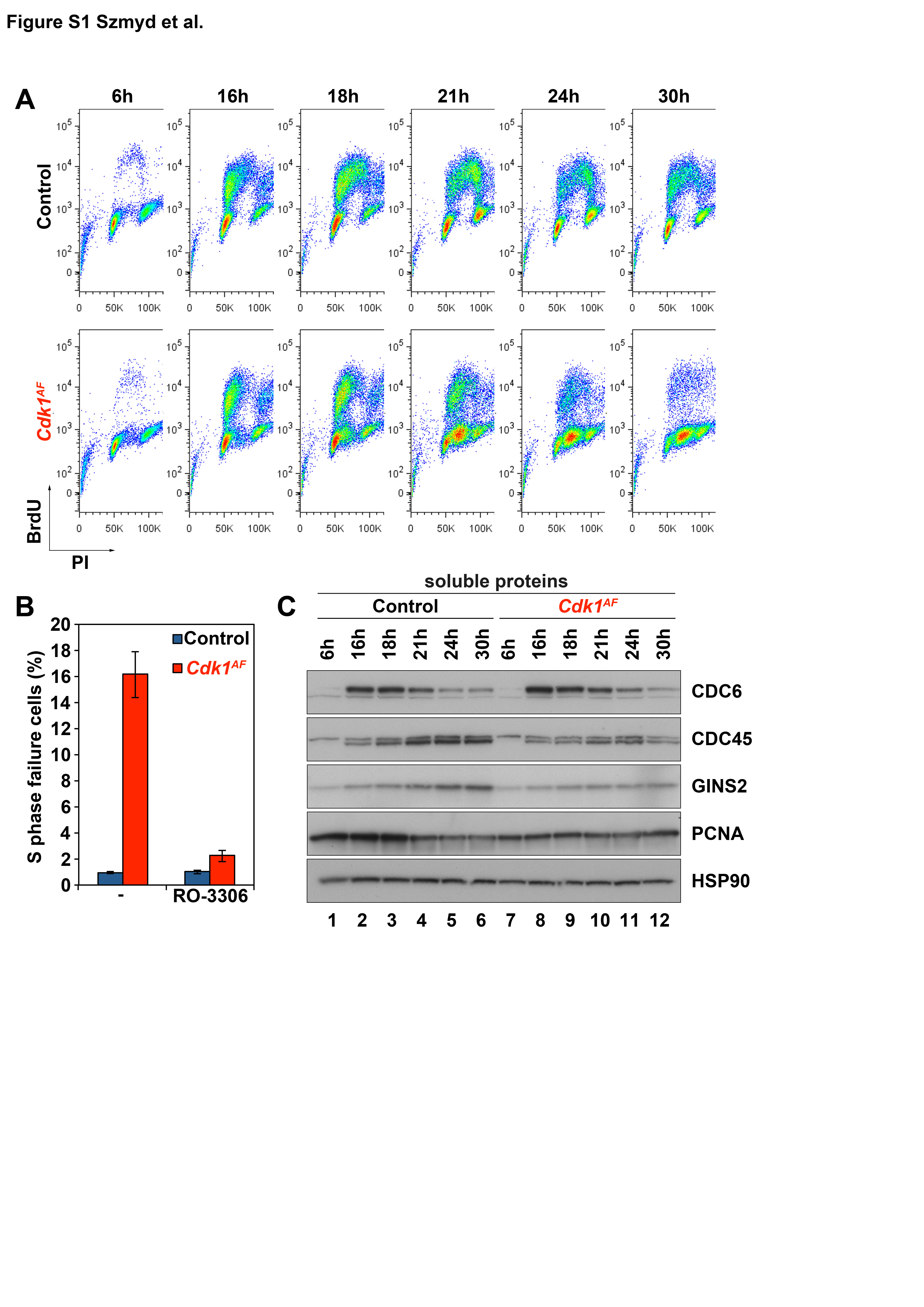

Supplement: Supplementary file 2 — Figure S1 [file 41388_2018_464_MOESM2_ESM.jpg]

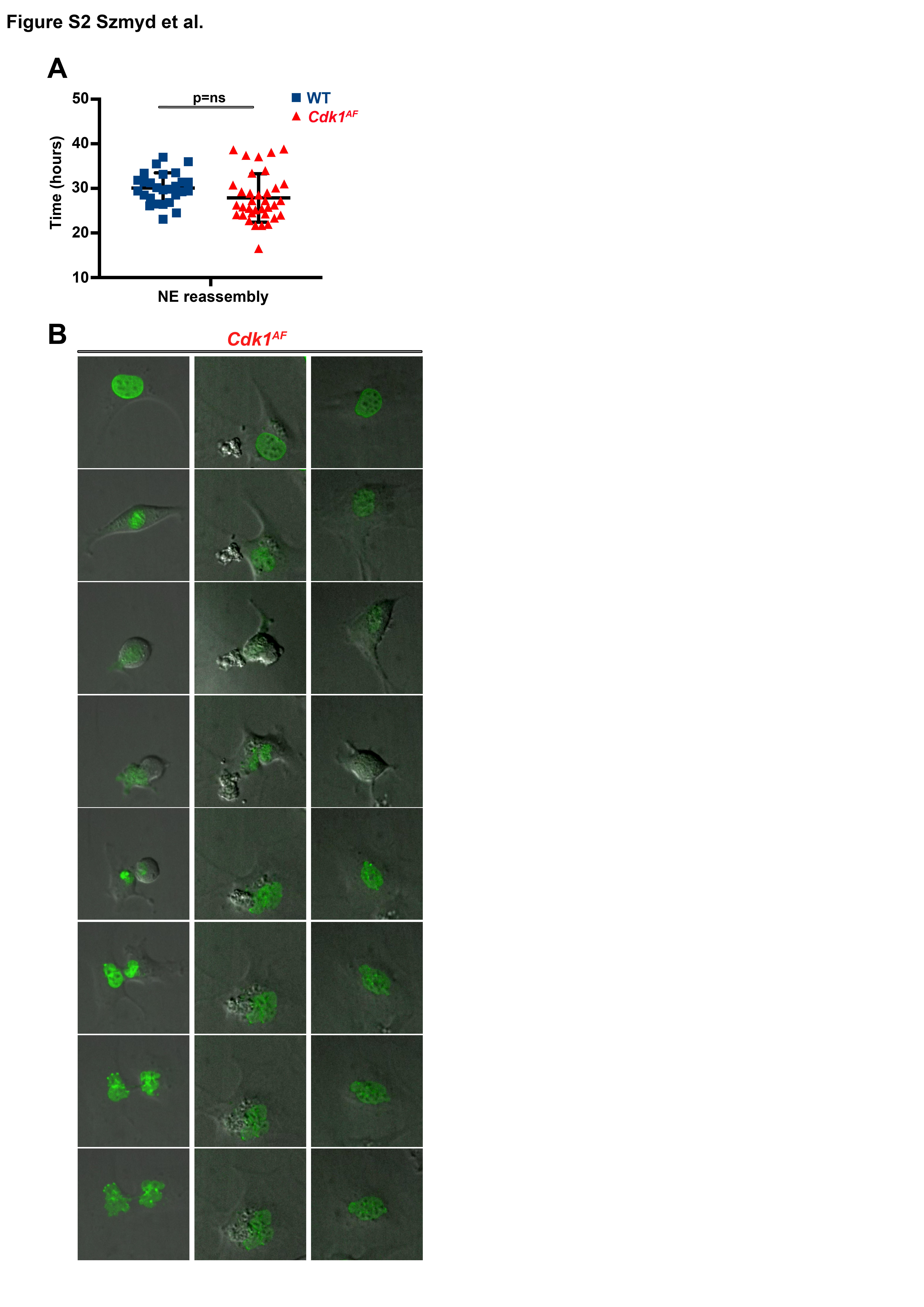

Supplement: Supplementary file 3 — Figure S2 [file 41388_2018_464_MOESM3_ESM.jpg]

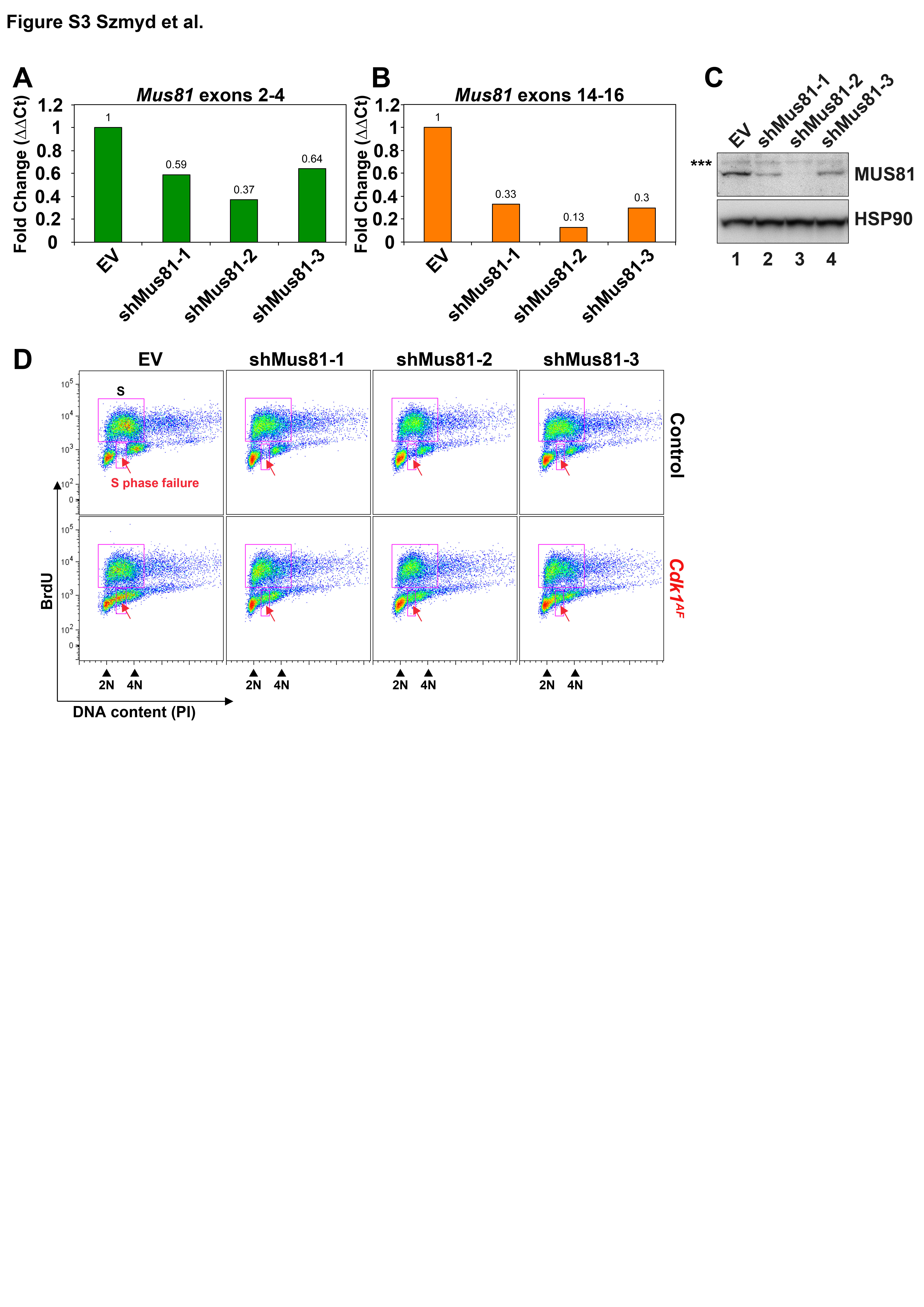

Supplement: Supplementary file 4 — Figure S3 [file 41388_2018_464_MOESM4_ESM.jpg]

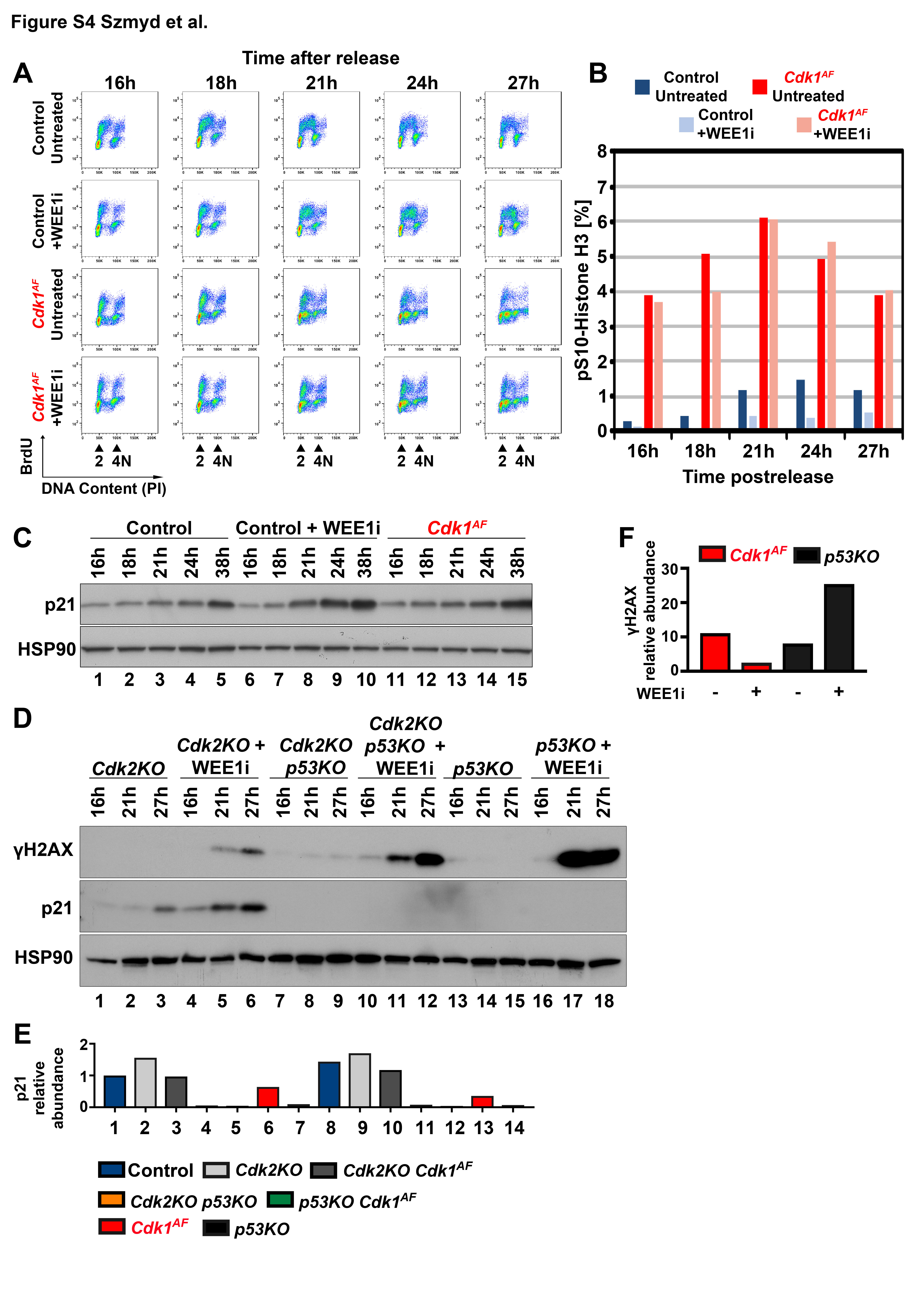

Supplement: Supplementary file 5 — Figure S4 [file 41388_2018_464_MOESM5_ESM.jpg]
